# Hypermethylation of *RAD9A* intron 2 in childhood cancer patients, leukemia and tumor cell lines suggest a role for oncogenic transformation

**DOI:** 10.17179/excli2021-4482

**Published:** 2022-01-07

**Authors:** Danuta Galetzka, Julia Böck, Lukas Wagner, Marcus Dittrich, Olesja Sinizyn, Marco Ludwig, Heidi Rossmann, Claudia Spix, Markus Radsak, Peter Scholz-Kreisel, Johanna Mirsch, Matthias Linke, Walburgis Brenner, Manuela Marron, Alicia Poplawski, Thomas Haaf, Heinz Schmidberger, Dirk Prawitt

**Affiliations:** 1 Department of Radiation Oncology and Radiation Therapy, University Medical Centre, Mainz, Germany; 2 Institute of Human Genetics, Julius Maximilians University, Würzburg, Germany; 3 Institute of Pathology, Julius Maximilians University, Würzburg, Germany; 4 Center for Pediatrics and Adolescent Medicine, University Medical Centre, Mainz, Germany; 5 Bioinformatics Department, Julius Maximilians University, Würzburg, Germany; 6 DRK Medical Centre, Alzey, Germany; 7 Institute of Clinical Chemistry and Laboratory Medicine, University Medical Centre, Mainz, Germany; 8 Division of Childhood Cancer Epidemiology, Institute of Medical Biostatistics, Epidemiology and Informatics, University Medical Centre, Mainz, Germany; 9 Department of Hematology, University Medical Centre, Mainz, Germany; 10 Federal Office of Radiation, Neuherberg, Germany; 11 Radiation Biology and DNA Repair, Technical University of Darmstadt, Germany; 12 Institute of Human Genetics, University Medical Centre, Mainz, Germany; 13 Department of Obstetrics and Women's Health, University Medical Centre, Mainz, Germany; 14 Leibniz Institute for Prevention Research and Epidemiology - BIPS, Bremen, Germany; 15 Institute of Medical Biostatistics, Epidemiology and Informatics, University Medical Centre, Mainz, Germany

**Keywords:** RAD9A, childhood cancer, hypermethylation, regular body cells, somatic mosaicism, leukemia, DNA repair

## Abstract

Most childhood cancers occur sporadically and cannot be explained by an inherited mutation or an unhealthy lifestyle. However, risk factors might trigger the oncogenic transformation of cells. Among other regulatory signals, hypermethylation of *RAD9A* intron 2 is responsible for the increased expression of RAD9A protein, which may play a role in oncogenic transformation. Here, we analyzed the *RAD9A* intron 2 methylation in primary fibroblasts of 20 patients with primary cancer in childhood and second primary cancer (2N) later in life, 20 matched patients with only one primary cancer in childhood (1N) and 20 matched cancer-free controls (0N), using bisulfite pyrosequencing and deep bisulfite sequencing (DBS). Four 1N patients and one 2N patient displayed elevated mean methylation levels (≥ 10 %) of *RAD9A*. DBS revealed ≥ 2 % hypermethylated alleles of *RAD9A,* indicative for constitutive mosaic epimutations. Bone marrow samples of NHL and AML tumor patients (n=74), EBV (Epstein Barr Virus) lymphoblasts (n=6), tumor cell lines (n=5) and FaDu subclones (n=13) were analyzed to substantiate our findings. We find a broad spectrum of tumor entities with an aberrant methylation of *RAD9A*. We detected a significant difference in mean methylation of *RAD9A* for NHL versus AML patients (p ≤0.025). Molecular karyotyping of AML samples during therapy with hypermethylated *RAD9A* showed an evolving duplication of 1.8 kb on Chr16p13.3 including the PKD1 gene. Radiation, colony formation assays, cell proliferation, PCR and molecular karyotyping SNP-array experiments using generated FaDu subclones suggest that hypermethylation of *RAD9A* intron 2 is associated with genomic imbalances in regions with tumor-relevant genes and survival of the cells. In conclusion, this is the very first study of *RAD9A* intron 2 methylation in childhood cancer and Leukemia. *RAD9A* epimutations may have an impact on leukemia and tumorigenesis and can potentially serve as a biomarker.

## Abbreviations

AML acute myeloid leukemia

B-ALL acute lymphocytic leukemia

CAF cancer-associated fibroblasts

DBS deep bisulfite sequencing

DMEM Dulbecco's modified Eagle's medium

EBV Epstein Barr virus

EMRs epimutation rates

FACS flow cytometric cell cycle

FANC Fanconi anemia

FBS fetal bovine serum

Gy Gray

HNPCC hereditary non-polyposis colon cancer

PBL plasmablastic lymphoma

RA refractory anemia

TS tumor suppressor

## Introduction

Compared to the aging population, cancer is rare among children and young adults, representing <1 % of all cancers. Children are usually not exposed to an unhealthy lifestyle or an adverse environment, and only 5-10 % of children with cancers carry predisposing germline mutations and therefore, most childhood cancers should occur sporadically (D´Orazio, 2010[18]; Kuhlen et al., 2019[42]; Sylvester et al., 2018[66]). Tumorigenesis is a multistep process, involving an accumulation of genetic and epigenetic changes in multiple genes resulting in both the inactivation of tumor suppressor (TS) genes and/or activation of oncogenes (Karakosta et al., 2005[39]). These changes are mostly caused by inaccurate repair of DNA lesions. Cells are endowed with various pathways capable of repairing DNA damage or causing senescence and cell death. The key player *p53* gene regulates the global cellular response to DNA damage, primarily by controlling transcription of its target genes (Aubrey et al., 2016[2]). Most hereditary forms of cancer are caused by germline mutations in tumor suppressor genes (frequently DNA repair genes), predisposing patients to tumor development, which itself is triggered by inactivation of the second TS allele (Knudson, 1996[40]). Accumulating evidence suggests that similar to germline mutations, constitutive epimutations involving soma-wide hypermethylation of tumor suppressor genes in normal body cells, can cause phenocopies of cancer syndromes such as hereditary non-polyposis colon cancer (HNPCC) as well as breast- and ovarian cancer (Böck et al., 2018[9]; Goel et al., 2011[27]). During each cell division, both the DNA sequence and its epigenetic modifications are transmitted to the daughter cells. The error rate for copying DNA methylation patterns during DNA replication is estimated to be 10-100 times higher than for non-replicating DNA (Bennett-Baker et al., 2003[6]; Horsthemke, 2006[35]). Therefore, rapidly dividing cells, e.g. stem cells during embryonal development and organogenesis, may be particularly vulnerable for acquiring methylation defects (Lopez-Lazaro, 2018[51]; Tomasetti et al., 2017[67]). Constitutive mosaic *BRCA1* epimutations are for example detected in 4-7 % of newborn females without germline *BRCA1* mutations.

While the underlying mechanisms are poorly understood, mosaic normal tissue *BRCA1* methylation is associated with a 2-3-fold increased risk of high-grade serous ovarian cancer (HGSOC) (Lønning et al., 2019[50]). For some cancer-predisposing genes, i.e. *MLH1*, *MSH2*, and *DAPK1*, the probability for *de novo* epimutations depends on cis-regulatory genetic sequence variants (Hitchins, 2016[33]; Ligtenberg et al., 2009[47]; Raval et al., 2007[63]). Furthermore, we have previously described monozygotic twin sisters discordant for childhood leukemia and a constitutive mosaic epimutation in the *BRCA1* TS gene (Galetzka et al., 2012[24]). We could show that affected fibroblasts phenotypically resemble cancer-associated fibroblasts (CAF) (Etzold et al., 2016[20]). Since constitutive epimutations usually occur in a mosaic state with variable proportions of affected cells in different tissues, they are most likely not transmitted through the germline but may arise during early development.

One possible explanation for the onset of sporadic childhood cancers is somatic mosaicism. The prenatal origin hypothesis postulates, that childhood cancers arise from postnatally persisting embryonal or more differentiated cells that carry predisposing mutations (Marshall et al., 2014[56]). Several studies have demonstrated prenatal oncogenic events underlying acute leukemia in childhood (Gale et al., 1997[23]; McHale et al., 2003[57]).

RAD9A has a dual role in cancer (lack of the protein causes cellular sensitivity to DNA damage and in turn provokes cancer, the abundance of the protein due to hypermethylation in intron 2 of the gene leads to advance cancer conditions in prostate and breast cancer). *Knock out or knockdown* of RAD9A in mammalian cells causes cellular sensitivity to a large variety of radiations and chemicals that damage DNA and influences genomic stability through complex and diverse activities (Tsai and Kai, 2014[68]). RAD9A is essential for maintaining genomic integrity even in the absence of exogenous DNA damaging agents, as loss of function causes increased frequencies of spontaneous chromosome and chromatid breaks, gene mutation and micronuclei formation (Hopkins et al., 2004[34]; Zhu et al., 2005[80]). Decreased *RAD9A* levels due to an RNA interference approach in prostate cancer cell lines dramatically reduce tumorigenicity in nude mouse xenografts, indicating not only that RAD9A has a critical, causal role in this type of cancer, but also suggesting that it plays a major role in DNA damage resistance and genomic integrity (Lieberman et al., 2017[45]; Zhu et al., 2005[80], 2008[79]). Importantly RAD9A functions as a pro-apoptotic or anti-apoptotic factor (Komatsu et al., 2000[41]; Zhu et al., 2005[80]). The pro-apoptotic function by RAD9A is mediated by a BH3-like domain in its N-terminal region that binds the anti-apoptotic proteins BCL-XL and BCL-2 thus promoting in this context programmed cell death (Lieberman et al., 2011[44]). The choice and the regulation of the two pathways are not known yet. Interestingly the methylation of three CpG's in intron 2 of the *RAD9A *gene was proven to influence the expression of the RAD9A protein. More than 45 % of prostate tumors have aberrantly high levels of RAD9A as a result of intron 2 hypermethylation and hypermethylation of this site was also found in breast cancer (Broustas et al., 2012[10]; Cheng et al., 2005[12]; Lieberman et al., 2018[46]). The current data indicates that both, the lack of or permanent overexpression of RAD9A may have harmful, tumor-promoting effects and the right balance of this important gene is the critical point in tumorigenesis.

Based on our previous expression studies (Weis et al., 2011[73]), here we investigated the epimutation rate of *RAD9A* in a unique cohort, consisting of fibroblasts derived from individuals who survived childhood cancer and subsequently developed a second primary cancer (2N) and matched (first tumor, manifestation age, sex) individuals with childhood cancer but without second cancer (1N). Moreover, fibroblasts of 20 (age- and gender-matched) cancer-free individuals served as controls (0N).

We found a subset of former cancer patients (mostly 1N) with *RAD9A* mosaic methylation of intron 2, which must be considered as relevant epimutations. To get a better understanding of the role of the *RAD9A* gene methylation in the oncogenic transformation we analyzed leukemia cancer samples, tumor cell lines and generated FaDu subclones with divergent methylation values of *RAD9A *and analyzed its influence on cell viability. Radiation, growth and chemotherapy did not affect methylation in intron 2 of *RAD9A*. Therefore, we assume that the aberrant methylation in *RAD9A* may be an important cell signal marker and may be useful for diagnostic purposes during therapy.

## Materials and Methods

### Patient samples and cell lines

With the help of the German Childhood Cancer Registry, 20 individuals who survived a childhood malignancy and then developed a second primary cancer (2N) and 20 carefully matched (first tumor, manifestation age, sex) individuals who survived a childhood cancer without developing a second malignancy (1N) were recruited for the KiKme study (Cancer in Childhood and Molecular Epidemiology) (Marron, 2017[55]). Twenty matched patients (sex and age) without cancer from the Department of Accident Surgery and Orthopedics served as controls (0N). Participants were informed in writing and verbally of the procedures and purpose of this study. Signed informed consent documents were obtained from both patients and healthy individuals after genetic counseling. This study was approved by the Ethics Committee of the Medical Association of Rhineland-Palatinate (no. 837.440.03 [4102]; no. 837.262.12 [8363-F]; and 837.103.04 [4261]).

All patients were followed up from primary cancer diagnosis to recruitment for this study several years after treatment. Patient characteristics can be found in Supplementary data Table 1. The corresponding tumor samples were not available.

Affymetrix array SNP analysis (Weis et al., 2011[73]) of 1N and 2N patients did not reveal detectable copy number variations in tumor suppressor genes (*BRCA1*, *BRCA2*, *RAD9A*, *TP53*, *NF1*) or oncogenes (*PTPN11*, *ETV6-RUNX1*, *TCF3-PBX1*). No pathogenic germline mutations in* TP53*, *RAD9A,* *BRCA1, or BRCA2* were identified by Sanger sequencing using the ACMG criteria (*TP53* Mutation Database (http://p53.iarc.fr/); https://www.ncbi.nlm.nih.gov/clinvar/ and https://www.lovd.nl/). *RB1* gene analysis in patient 1N20 did not reveal a pathogenic mutation. Using bisulfite pyrosequencing, none of the matched (1N or 2N) childhood cancer patients of this study showed *BRCA1* hypermethylation, consistent with an epimutation (Galetzka et al., 2012[24]). Cell lines 0N24 and 2N24 were derived from a pair of discordant monozygotic twins. One twin suffered from childhood leukemia and later on from thyroid carcinoma, whereas her sister is completely healthy to date.

Bone marrow samples (excess material from routine chromosome diagnostics) were obtained from 27 patients with NHL (19 males, 8 females, aged 58.0±12.0 years), 27 AML (16 males and 11 females, aged 60.0±11.0 years), one pre-B-ALL (46,XY,t(9;22)(q34;q11.2)[2]/46,XY[25], one AML (46,XY,der(7)(q-).ish del(16)(q22)[12]/ 46,XY[10], and one plasmablastic lymphoma (PBL) with complex aberrations in 60 % of cells, consisting of a hypodiploid clone (28 %) and a hyperdiploid line (32 %). The analysis of the samples was approved by the Ethics Committee of the Medical Association of Rhineland-Palatinate (no. 2019-14677).

The FaDu tumor cell line (from hypopharyngeal carcinoma), carrying mutations in *CDKN2A* (c.151-1G>T), *TP53* (c.376-1 G>A; c.743G>T) and *SMAD4* (c.1_1659del1659) was purchased from ATCC and characterized using SNP-array analysis in 24.05.18. The cell line BT-549 (from ductal carcinoma) was purchased from CLS (Eppelheim, Germany). The cell line, EFO21 (from ovarian serous cystadenocarcinoma), was purchased from DSMZ (Braunschweig, Germany), the cell lines MCF7 (from breast carcinoma) and T47D (from breast cancer) from ATCC (Manassas, VA, USA). All cell lines were verified using the STR (Short Tandem Repeat)-analysis (DMSZ German Collection of Microorganisms and Cell Cultures GmbH on 21.11.2018). All the subsequent research analyses were carried out in accordance with the approved guidelines and regulations.

### Cell culture

The generation of primary fibroblasts from skin biopsies is described in Supplementary data Methods). The primary fibroblasts cell lines were cultured in Dulbecco`s Minimal Essential Medium with Earle's salts DMEM (Invitrogen, Karlsruhe, Germany), supplemented with 15 % fetal bovine serum (FBS) (Merck, Darmstadt, Germany), 1 % vitamins and 1 % antibiotics (penicillin/streptomycin) (Biochrom, Berlin, Germany) in a 90 % humidified incubator with 5 % CO_2_ at 37 °C.

The FaDu tumor cell line was cultured in Dulbecco`s Minimal Essential Medium (Sigma-Aldrich, St. Louis, USA) containing 1 % non-essential amino acids (Biochrom, Berlin, Germany), 15 % FBS (Biochrom, Berlin, Germany) and 1 % antibiotics (Biochrom, Berlin, Germany). Passaging was done using 0.05 % trypsin with 0.1 % ethylene-diamine-tetra-acetate (EDTA) (Biochrom, Berlin, Germany). FaDu derived single-cell clones were generated by limiting dilution of the primary cell line and propagated in DMEM (Invitrogen, Karlsruhe, Germany), supplemented with 15 % FBS, 1 % vitamins and 1 % antibiotics (Biochrom, Berlin, Germany) using 96-well plates (Cellstar, Greiner GmbH, Kremsmünster, Germany). After 48 h wells with only one cell were selected via microscopic examination. Subsequent propagation was performed in conditioned medium (one day old medium of primary culture at 50 % confluency, sterile filtered using 0.2 µm filter). The cells were transferred when they reached 80 % confluency, initially into a 24-well plate, later in a 6-well and finally in the 10 cm petri dishes.

Cell lines (MCF7, BT-549, EFO21 und T47D) were cultivated in RPMI1640 (Gibco, Life Technologies, Darmstadt, Germany) supplemented with 2.5 % HEPES buffer (Sigma, Darmstadt, Germany) and 1 % antibiotics (Life Technologies, Darmstadt, Germany). BT-549, MCF7 and T47D were additionally supplemented with 10 % FBS, EFO21 with 20 % FBS.

All experiments using the primary fibroblasts were performed with growth-arrested cells in the G0/G1 stage. Confluency was achieved by contact inhibition and subsequent cultivation for two weeks and confirmed by FACS (flow cytometric cell cycle) analysis. For comparisons of 2N, 1N, and 0N patients, fibroblasts with a similar passage number 5 (±2) were used.

To obtain proliferating immortal lymphoblastoid cells Epstein Barr virus (EBV) transformation of resting B cells (in peripheral blood lymphocytes) is widely used (Neitzel, 1986[59]). Lymphoblastoid cells were generated in our routine labor and harvested in an early passage after stable infection.

### Daunorubicin treatment

Fibroblasts were cultured as described above. The cells were cultured in T25 flasks to 80-90 % confluency, then a non-lethal dose of 3 µM daunorubicin (Pfizer Pharma PFE GmbH, Berlin, Germany) (Przybylska et al., 2001[60]) was added. After two hours the medium was replaced 2, 4 h and 24 h post-treatment and the cells were harvested using trypsin. Quantification of γH2AX was performed as described previously (Weis et al., 2008[72]).

### Growth kinetics of FaDu and subclones

Tumor cell lines were cultured as described above. Cells were seeded at a density of 5*10^4^ in T75 cell culture flasks in triplicates. The cells were harvested at different time points using trypsin and total cell numbers were determined using the Moxi^z^ automatic cell counter (Orflo, Ketchum, USA) at every time point. The cellular proliferation rate was calculated as the cumulative population doublings (CPD). The statistical analysis was done using the linear mixed-effects model fit by REML setting the biological replicate as a random variable.

### Irradiation

Cells were exposed to X-rays with a D3150 X-Ray Therapy System (Gulmay Ltd, Surrey, UK) at 140 kV and a dose rate of 3.62 Gy/min at room temperature. Sham irradiated control cells were kept at the same conditions in the radiation device control room. The FaDu tumor cell line was irradiated at 80 % confluency. Cells were exposed to single doses ranging from 2-8 Gy and harvested at 15 min, 2 h, and 24 h after irradiation. For fractionalized irradiation, fibroblasts were irradiated 4x, 8x, or 10x with doses of 2 Gy and 4 Gy, within 20 days. Cells were given one day of recovery time between exposures and the medium was changed twice a week. Cells were harvested one day after the final irradiation.

### Colony formation assay

Clonogenic survival was determined in colony formation assays adapted after Menegakis et al. (2009[58]). At passage p8, cells were seeded (1x10^5^) in 10 cm diameter Petri dishes in triplicates. After five days and one medium change, the cells were irradiated with 2 Gy, 4 Gy, 6 Gy, and 8 Gy. Sham irradiated cells were kept at the same conditions in the radiation device control room (0 Gy). After 24 h cell suspensions were obtained for each dose and different seeding densities were plated in triplicates. The remaining cells were pelleted, washed with PBS and stored at -80 °C for further experiments. 14 days after irradiation colonies were fixed and stained with crystal violet. The image of a FaDu cell line (0 Gy) was made with the ZEISS Axiovert microscope using the Clone software at 10x magnification. The grayscale transformation was done with GIMP 2.10.14 software. Colonies defined as >50 cells were counted and surviving fractions were expressed in terms of plating efficiency. Survival data after radiation were fitted to linear quadratic regression models employing the maximum likelihood approach (R package CFAssay). Differences between curves were evaluated using the F-test. Adjustment of p-values was done using the method of Benjamini and Hochberg (1995[5]).

### Flow cytometry

Cells were washed with PBS (37 °C; 1 ml/25 cm^2^) and trypsinized (Trypsin/EDTA (37 °C; 1 ml/ 25 cm^2^), for 5 min at 37 °C. The reaction was stopped by the addition of a two-fold volume of culture medium at 37 °C and cells were collected by centrifugation at 300 x g for 6 min. 70 % ethanol (-20 °C) was added dropwise to the cell pellet under permanent turbulence (~ 4 ml/10^5^ cells) and the suspension was stored for ≥ 30 min at 4 °C or overnight. Later, the cell suspension was centrifuged at 300 x g for 6 min, and the supernatant was discarded. Cells were re-suspended in PBS (without magnesium and calcium -/-) with RNase (20 µg/ 10^4^ cells) and incubated for 30 min at 37 °C. After an additional centrifuge step of 300 x g for 6 min, cells were re-suspended in HOECHST 33258-staining solution (0.2 µg/ml in 1x PBS-/-) (~ 4 ml/ 10^5^ cells) and again incubated for ≥ 30 min at 4 °C. The analysis was performed using FACS Canto II (BD Biosciences, Germany).

## Molecular Analysis Methods

### Bisulfite pyrosequencing

Genomic DNA was isolated with the NucleoSpin tissue kit (Macherey-Nagel, Germany). Bisulfite conversion of 0.2 µg DNA was performed with the EpiTect Bisulfite Kit (Qiagen, Germany) according to the manufacturer's instructions. PCR and sequencing primers for the genes mentioned above were designed with PyroMark Assay Design 2.0 software (Qiagen, Germany) (Supplementary information Table 1). The 50 µl PCR reactions consisted of 25 µl Pyro PCR Mix 2x, 5 µl Coral load 10x, 1 μl of each forward and reverse primer (10 µM), 17 μl PCR-grade water and 1 μl (~100 ng) bisulfite-converted template DNA (PyroMark PCR Master Mix, Qiagen). PCR amplifications were performed with an initial denaturation step at 95 °C for 5 min, 35 cycles (95 °C for 30 sec, TM (gene analyzed) for 45sec and 72 °C for 45 sec) and a final extension step at 72 °C for 10 min. Bisulfite pyrosequencing was performed on a PyroMark Q96 MD Pyrosequencing System using the PyroMark Gold Q96 CDT Reagent Kit (Qiagen, Germany) and 1 µl of sequencing primers (10 µM). Data analysis was performed with the Pyro Q-CpG software (Qiagen, Germany). Single CpG errors which arise presumably due to bisulfite conversion errors (technical variation) were in the order of 1 %. This value was calculated by analyzing samples of three patients in three replicates.

Data were analyzed using the Kruskal-Wallis rank sum-test. P-values were adjusted for multiple testing using the method of Benjamini and Hochberg (1995[5]). Analysis of the AML and NHL patient data was performed using the Mann-Whitney-U Test (RV3.6.2)

### Deep bisulfite sequencing (DBS)

Next-generation sequencing (NGS) libraries for DBS were generated as described previously (Böck et al., 2018[9]). PCR amplification of *APC*, *CDKN2A*, *TP53*, and *RAD9A* was performed using primers containing a target-specific part and partial adapter overhangs (Supplementary informatin Table 2). Following the purification of the amplicons with magnetic beads (0.9:1), the DNA concentration was determined with the dsDNA BR Assay System (Life Technologies, USA). Samples were diluted to 0.2 ng/µl in a total volume of 15 µl each. After pooling, barcoding was performed by PCR with NEBNext Multiplex Oligos for Illumina, Dual Index Primer Set 1 (New England BioLabs, Germany), followed by another bead cleaning step (0.9:1). DNA concentration and fragment length were determined with the High Sensitivity DNA kit on a 2100 Bioanalyzer (Agilent Technologies, USA). All barcoded pools were diluted to 2 nM and pooled to the final library. Denaturation and preparation of the library and PhiX control were done according to the manufacturer's protocol (Illumina, USA). Paired-end sequencing was performed on an Illumina MiSeq using the MiSeq Reagent Kit v2 (2 x 150 cycles) cartridge.

The sequences in FASTQ format were processed using the Amplikyzer2 pipeline (Rahmann et al., 2013[62]), which provides a detailed nucleotide-level analysis including the calculation of CpG methylation rates. All sequences were aligned to the genomic sequence of each amplicon using default settings. For the subsequent extraction of reads and CpG-wise methylation status only reads with an overall bisulfite conversion rate of over 95 % were considered. Further downstream processing of Amplikyzer2 output files and subsequent analyses of methylation rates were performed using R-scripts. Statistical analyses were performed with the statistical software package R (Version 3.2.2) (R-Core Team).

### SNP array analysis (Karyotyping)

High-resolution screening for microdeletions and duplications of the FaDu cell line and its subclones was performed with the Affymetrix GeneChip Genome-Wide Human SNP Cyto HD, following the protocol developed by the manufacturer (Affymetrix, Santa Clara, CA, USA). Data calculation was performed with Chromosome Analysis Suite 3.1.0.15 and NetAffx Build 33.1 (hg19). Karyotypes were described in accordance with the International System for Human Cytogenetic Nomenclature (ISCN2013).

### PCR and sequencing

Genomic DNA was isolated using the NucleoSpin tissue kit (Macherey-Nagel, Germany). PCR reactions were performed using the FastStart Taq DNA Polymerase, dNTPack, kit # 04738403001 (Roche Diagnostics, Germany). PCR was performed in three stages with one cycle of 5 min at 94 °C, forty cycles (15 sec, 94 °C, 30 sec primer TM, 1 min 68 °C) and one cycle of 2 min at 72 °C, using 30 ng DNA per reaction. Primers were designed using NCBI's primer designing tool (https://www.ncbi.nlm.nih.gov/tools/primer-blast/). The following primer pairs were used; forward primer (AGGCAGTCAGTCGGAAAGTG) and reverse (TTGGAACCTGCTGATTCGCT) for *CHD2* (NM_001271) TM 62 °C; forward primer (ACAGCTGTTCTCACGGAAGG) and reverse (TAGGCTGCTGAGGATGGTCT) for *SPATA8* (NR_158221) TM 62 °C and forward primer (GCAAGAAGAAGATGAAGAGC) and reverse (GGCAGGTAAGCTCAGGTTTT) for *SMARCA1* (NM_003069) TM 64 °C. Sequence reactions were performed by Genterprise Germany, using PCR products digested with Exo/SaP (New England Biolabs, Germany). Uncropped image may be found in Supplementary data Figure 5.

### RT-qPCR expression analysis of RAD9A

Total RNAs were prepared from three independent cell culture samples (growth kinetics experiment) using the Nucleo Spin RNA Plus Kit from Macherey-Nagel (MN). 2 µg of the RNA samples were reversely transcribed into cDNA using the SuperScript IV First-Strand random hexamer Synthesis System (Invitrogen).

Forward and reverse primers (Exon-spanning for gene expression) were designed with the Primer-Blast program (https://www.ncbi.nlm.nih.gov/tools/primer-blast/). The *TBP* gene was used as an endogenous control. The following primer pairs (5´-3´ orientation) were used; forward primer (CGGCAACGTGAAGGTGCTC) and reverse (CAGGTTGTGAGTCTTCCGCA) for *RAD9A *(NM_004584.3) TM 65°C; forward primer (CCACTCACAGACTCTCACAAC) and reverse (CTGCGGTACAATCCCAGAACT) for *TBP* (NM_003194.5)

Each 10 µl reaction volume contained 40 ng cDNA in 5 µl Sybr-Green Master Mix (Biozym), 2 µl RNase-free PCR graded water (MN), 1 µl each of forward and reverse primer (10 µM). All reactions were performed in triplicate and two stages, with one cycle of 95 °C for 10 min (first stage) and 45 cycles of 94 °C for 10 s, (TM-primer)°C for 10 s, and 72 °C for 10 s (second stage) using the LightCycler 480II Roche. Amplification qualities were assayed using melting curves and agarose gel analysis. The qPCR amplification efficiency was calculated using the LinReg program (Ruijter et al., 2014[64]) and the CT values were corrected using the mean amplification efficiency. Relative quantification was carried out with the ΔΔCT method using the endogenous control gene for calibration. The student *t*-test was used for the comparison of data between two or more groups. All tests were two-tailed; p<0.05 was considered statistically significant.

## Results

### Epimutation analysis of fibroblasts derived from childhood cancer patients using bisulfite pyrosequencing and deep bisulfite sequencing

**Figure 1:**
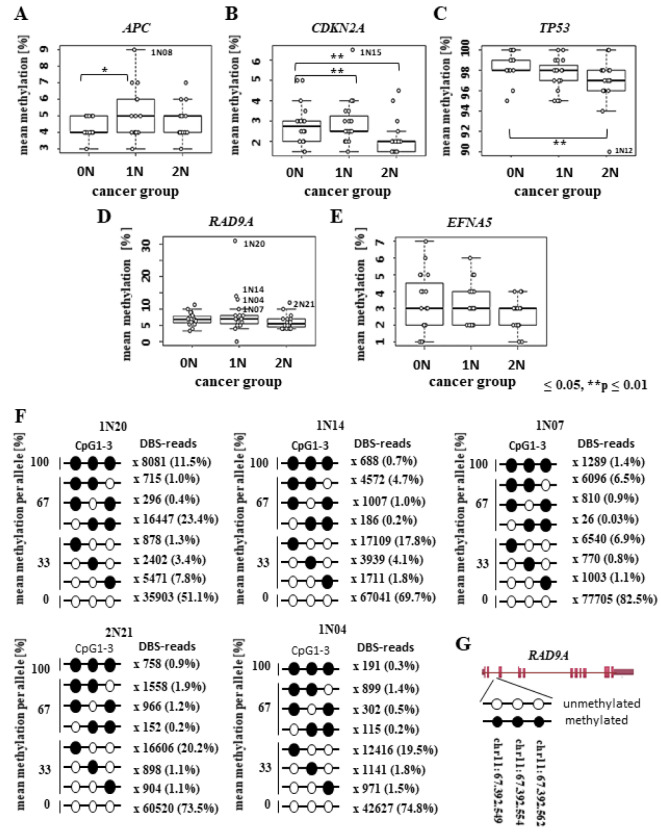
Methylation analysis of *RAD9A* and various TS genes using bisulfite pyrosequencing and deep bisulfite sequencing analysis (DBS) in childhood cancer patients. Box plots show the distribution of (A) *APC, *(B)* CDKN2A, *(C) *TP53,* (D) *RAD9A *and (E) *EFNA* mean methylation values (%) in 20 healthy controls (0N), 20 one-cancer (1N), and 20 two-cancer (2N) patients including outliers which are also listed in Table 1 using bisulfite pyrosequencing. Bars extend from the boxes to at most 1.5 times the height of the box. Statistical comparison was performed using the Kruskal-Wallis rank-sum test. *p ≤ 0.05, **p ≤ 0.01. (F) Four conspicuous 1N (1N20, 1N14, 1N07, 1N04) and one 2N (2N21) patients were analyzed using DBS and the results of *RAD9A* allele mean methylation values (%) are shown in lollipop diagrams for each patient. Black filled circles indicate 100 % methylation. The line numbers represent the DBS reads obtained for each CpG combination. (G) Position of CpG 1-3 in *RAD9A *on chromosome 11.

In a previous study, we detected expression changes of RAD9A protein in fibroblasts of former childhood tumor patients with the prevalent cancer types being leukemias and solid tumors (Weis et al., 2011[73]). Alteration of gene expression may be caused by methylation changes of promotor or other regulation sites. The methylation of three CpG's in intron 2 of the *RAD9A* gene (Figure 1G(Fig. 1)) is known to regulate the expression of the RAD9A protein (Cheng et al., 2005[12]). Therefore, we analyzed the methylation of this site and as well as a panel of control genes (*APC, CDKN2A, EFNA5, TP53*) which were reported to be aberrantly methylated in related tumors and/or have a role in childhood malignancy (for further information see Supplementary material, Supplementary Table 1) using bisulfite pyrosequencing and deep bisulfite sequencing (DBS).

### Bisulfite pyrosequencing reveals five patients with increased RAD9A intron 2 mean methylation

We determined the mean methylation of the promoter regions in *APC* (NC_000084.6), *CDKN2A* (NC_000009.12), and *EFNA5* (NC_000005.10), a cis-regulatory region in *RAD9A* intron 2 (NC_000011.10), and a mutation hotspot in *TP53* exon 6 (NC_000017.11) in 20 primary fibroblast cell lines of cancer-free controls 0N and matched patients 1N and 2N (Figure 1A-E(Fig. 1)). There were significant variations in methylation among the groups. *APC* (Figure 1A(Fig. 1)) proved to be hypermethylated in 1N in comparison to the 0N control (adj.p = 0.03). *CDKN2A* (Figure 1B(Fig. 1)), which is often mutated in a variety of cancers, exhibited hypermethylation in the 1N group (adj.p = 0.006), while it was hypomethylated in 2N group in comparison to 0N group (adj.p = 0.008). The 2N group showed a hypomethylation in *TP53* (Figure 1C(Fig. 1)) in comparison to the control group 0N (adj.p = 0.01). No significant differences between the groups were detected for *RAD9A* genes (Figure 1D(Fig. 1)) and* EFNA5* (Figure 1E(Fig. 1)). However, we identified several patients with aberrant methylation values. Patients 1N08 and 1N15 exhibited promoter hypermethylation of *APC* (9 %) and *CDKN2A* (7 %), respectively, whereas patient 2N12 displayed hypomethylation (95 %) of the *TP53* mutation hotspot. Five patients (1N04, 1N07, 1N14, 1N20, and 2N21) showed increased *RAD9A* intron 2 mean methylation, ranging from 10 % to 31 % (Figure 1A-D(Fig. 1)).

### Analysis of aberrant methylation using deep bisulfite sequencing (DBS) shows likely pathogenic epimutation rates in four childhood cancer patients

Previously, we have demonstrated that samples with aberrant methylation determined by bisulfite pyrosequencing can be considered as likely candidates for mosaic epimutations, which may have a role in carcinogenesis (Galetzka et al., 2012[24]). Therefore, we focused on patients with *RAD9A* hypermethylation.

The average methylation changes could be due to either single CpG methylation errors at different positions in a large number of alleles or due to allele methylation errors, where most CpGs in individual DNA molecules are aberrantly methylated. Because it is usually the density of CpG methylation in a cis-regulatory region rather than individual CpGs that turns a gene "on" or "off" (Weber et al., 2007[71]), allele methylation errors must be considered putatively as functionally relevant epimutations. To investigate the allele positions of the methylated CpG's, we performed an analysis utilizing deep bisulfite sequencing (DBS), which can determine the methylation profiles of many thousands of individual DNA alleles for multiple genes and samples in a single experiment and thus directly measure epimutation rates (EMRs). To determine the density of CpGs and allele methylation ratio in the present study, we performed DBS on the patients with presumed epimutations (Figure 1A-E(Fig. 1) and Table 1(Tab. 1)). Alleles with >50 % aberrantly (de)methylated CpGs in DBS are considered as functionally relevant epimutations.

Consistent with an epimutation screen in breast cancer susceptibility genes (Böck et al., 2018[9]), we considered EMRs >1 % as elevated and EMRs >2.5 % as likely pathogenic constitutive epimutations. Using the above-described classification, we did not detect any *APC* epimutations in fibroblasts of patient 1N08. Patient 1N15 displayed 0.2 % EMRs in *CDKN2A *and patient 2N12, 0.3 % EMRs in *TP53*. Five patients with suspected *RAD9A* epimutations displayed 2.0 % to 24.5 % EMRs. Four childhood cancer patients (1N20, 1N14, 1N07 and 2N21) showed* RAD9A* epimutations and childhood cancer patient 1N04 showed an elevated *RAD9A* EMR. Overall, 10 % (4 of 40) childhood cancer patients (1N and 2N) had *RAD9A* epimutations in their fibroblast cells (Table 1(Tab. 1) and Figure 1F(Fig. 1)).

### RAD9A hypermethylation may indicate compromised integrity of important genes

**Figure 2:**
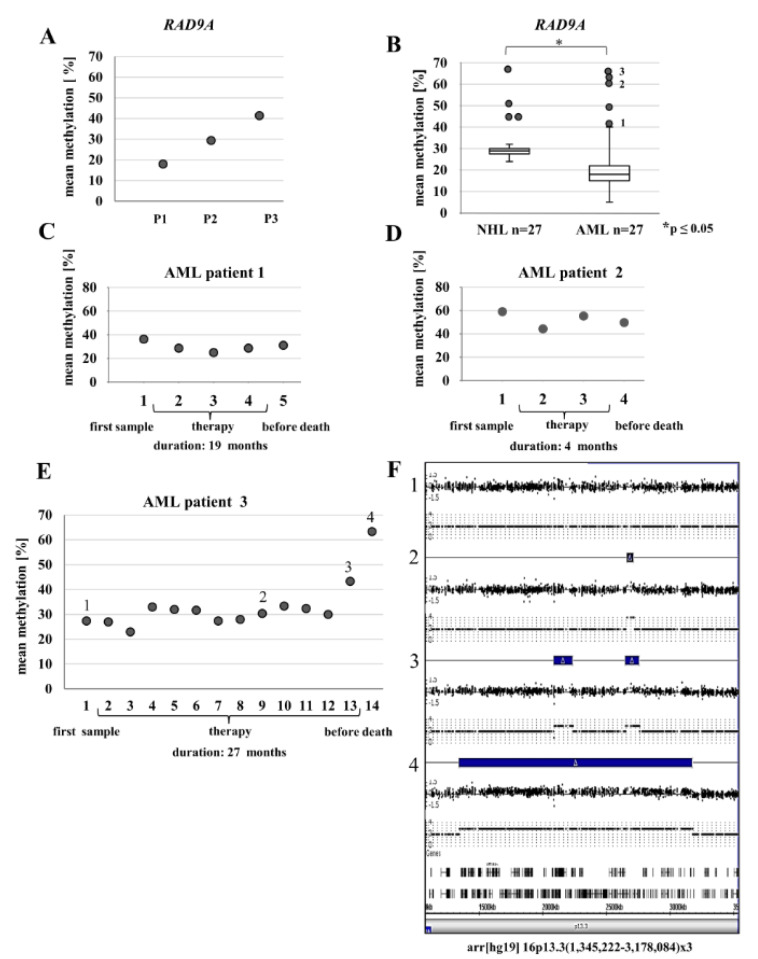
*RAD9A* methylation in the bone marrow (BM) of leukemia patients and BM samples of selected AML patients during therapy. (A) Patient (P1) with pre-ALL and a Philadelphia chromosome in <10% of analyzed bone marrow cells displayed mean methylation of 18%. Patient (P2) with AML and 50% bone marrow cells with abnormal karyotype displayed 29% *RAD9A* methylation. Patient (P3) with plasmablastic lymphoma and 60% cells with complex aberrations show 41 % *RAD9A *methylation. (B) Analysis of 27 NHL and 27 AML bone marrow samples revealed overall mean methylation of 30 % for NHL and 20 % for the AML patients. In a few patients, the mean methylation was elevated. (C-E) Methylation profiles of *RAD9A* in 3 AML patients during therapy. Please note changes in methylation of *RAD9A*. (F) Using a genome-wide SNP array four bone marrow samples of the AML patient 3 (1-4) were analyzed. Corresponding images from chromosome 16p13.3 display a progressive duplication of the 16p13.3(1,345,222-3,178,084) fragment in the last 3 samples (blue bars). The duplicated region contains 106 genes of which 9 genes are involved in cancer (*UBE2I, NUBP2, IGFALS, NTHL1, TSC2, PKD1, PDPK1, TCEB2, and TNFRSF12A*). For a larger version of this graph please see Supplementary information Figure 2E and F.

Four childhood patients with *RAD9A* EMR >2 % suffered from leukemia (ALL and Hodgkin lymphoma). To corroborate this finding, we first analyzed *RAD9A* methylation in the bone marrow of three leukemia patients. Patient P1 with pre-ALL and a Philadel-phia chromosome in <10 % of analyzed (bone marrow) cells displayed a mean *RAD9A* methylation of 18 %. Patient P2 with AML and 50 % bone marrow cells with abnormal karyotype displayed 29 % *RAD9A* mean methylation whereas patient P3 with PBL and 60 % cells of his bone marrow cells showed complex aberrations had a *RAD9A* mean methylation of 41 % (Figure 2A(Fig. 2)). The TCGA data base collection makes it possible to compare the expression of *RAD9A* in different tumor entities. In most tumor types *RAD9A* expression is deregulated (http://firebrowse.org/viewGene.html?gene=RAD9A) in comparison to controls. As leukemia and NHL occurred in our patients (primary secondary tumor) we conducted an extended analysis in leukemia tumor samples.

Further analysis of bone marrow samples from 27 NHL and 27 AML patients revealed a mean *RAD9A* methylation of 30 % for NHL and 20 % for the AML patients (p ≤0.025). We detected four NHL and five AML patients with elevated mean methylation levels, ranging from 38 % to 66 % in NHL and from 14 % to 63 % in AML patients (Figure 2B(Fig. 2)). We were able to analyze several bone marrow samples taken in the course of therapy for three AML patients (Figure 2C-E(Fig. 2)). The first bone marrow sample of AML patient 1 at diagnosis showed 36 % mean methylation of the *RAD9A* intron 2 sites. In course of the therapy, (life period: 19 months from diagnosis to death), the methylation levels decreased to 25 % and shortly before death increased to 30 % (Figure 2C(Fig. 2)). AML patient 2 had a rather high mean methylation of 59 % in the first bone marrow sample. Again, we could observe a slight decrease of methylation values (mean 44 %) during the therapy, but one month later the values increased again (mean methylation 55 %) and shortly after the last sample with the mean methylation value of 50 % was taken the patient deceased (life period from diagnosis to death was 4 months) (Figure 2D(Fig. 2)). The first bone marrow sample of AML patient 3 displayed the mean *RAD9A* intron 2 methylation values of 27 %. These values did slightly vary during the therapy. However, in a short period of 2 months before the patient died, he displayed strong hypermethylation of 43 %. This methylation further increased to 63 % (the life period from diagnosis to death was 27 months) (Supplementary data Figure 2).

In addition to *RAD9A *methylation, we analyzed bone marrow samples of this patient using a genome-wide SNP array for molecular karyotyping. As shown in Figure 2E(Fig. 2) the analyzed samples display a progressive duplication of the 16p13.3(1,345,222-3,178,084) fragment in the last 2 months before death. The final duplicated region contains 106 genes of which 9 genes are known to be involved in cancer (*UBE2I, NUBP2, IGFALS, NTHL1, TSC2, PKD1, PDPK1, TCEB2, and TNFRSF12A*).

To substantiate the connection between alteration of important genes and the hypermethylation of *RAD9A*, we examined two primary fibroblast cell lines known to have homozygous mutations either in *BRCA2* (*FANCD1*) or in *SLX4* (*FANCP1*) in contrast to primary fibroblast cell cultures (N=20, 0N). As expected, fibroblast control cells exhibited *RAD9A* mean methylation values ranging from 3-11 %, in contrast to *FANCD1* (28 %) and *FANCP1* (47 %) (Supplementary data Table 2).

### RAD9A methylation changes upon EBV transformation

**Figure 3:**
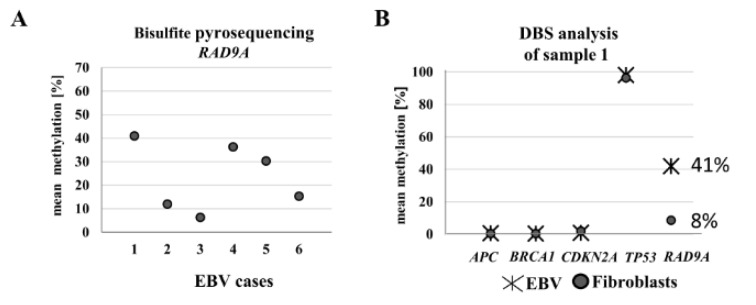
Analysis of *RAD9A* methylation in EBV-transformed lymphoblasts using bisulfite pyrosequencing and DBS. (A) The mean methylation varied in six different EBV-transformed lymphoblast cell lines from 6 % to 41 %. (B) The DBS analysis of the EBV cell line with the highest methylation value exhibited 41 % *RAD9A *methylation. In contrast, the methylation patterns of *APC, BRCA1, CDKN2A*, and *TP53* in this cell line remained virtually unchanged after transformation.

According to Cheng and colleagues (Cheng et al., 2005[12]), in breast cancer, *RAD9A* becomes an oncogene via hypermethylation in intron 2, due to consequently increased RAD9A transcription. We hypothesized that *RAD9A* hypermethylation is linked to the oncogenic transformation of a cell. EBV transformation of B cells results in unlimited growth and has been associated with particular forms of cancer, such as Burkitt's lymphoma, Hodgkin's lymphoma, nasopharyngeal carcinoma, and gastric cancer (Vockerodt et al., 2015[69]). Thus, EBV infection is now widely used to generate immortal lymphoblastoid cell lines. Global changes in DNA methylation may contribute to the pathogenesis of EBV (Hernandez et al., 2017[31]). We, therefore, tested EBV transformed lymphoblasts for changes in methylation of *RAD9A* in intron 2 using bisulfite sequencing. The mean methylation in six different EBV transformed cell lines varied from 6 % to 41 %, the corresponding fibroblast cells showed 8 % mean methylation in *RAD9A* (Figure 3B(Fig. 3)). The DBS analysis of the cell line with the highest mean methylation value (41 %) exhibited 9 % fully methylated alleles. In contrast, the methylation patterns of *APC*, *BRCA1*, *CDKN2A *(hypomethylated), and *TP53* (hypermethylated) remained virtually unchanged in this cell line after EBV transformation (Figure 3B(Fig. 3)).

### The parental FaDu and subclone cell lines are genetically diverse and display varying RAD9A methylation values

**Figure 4:**
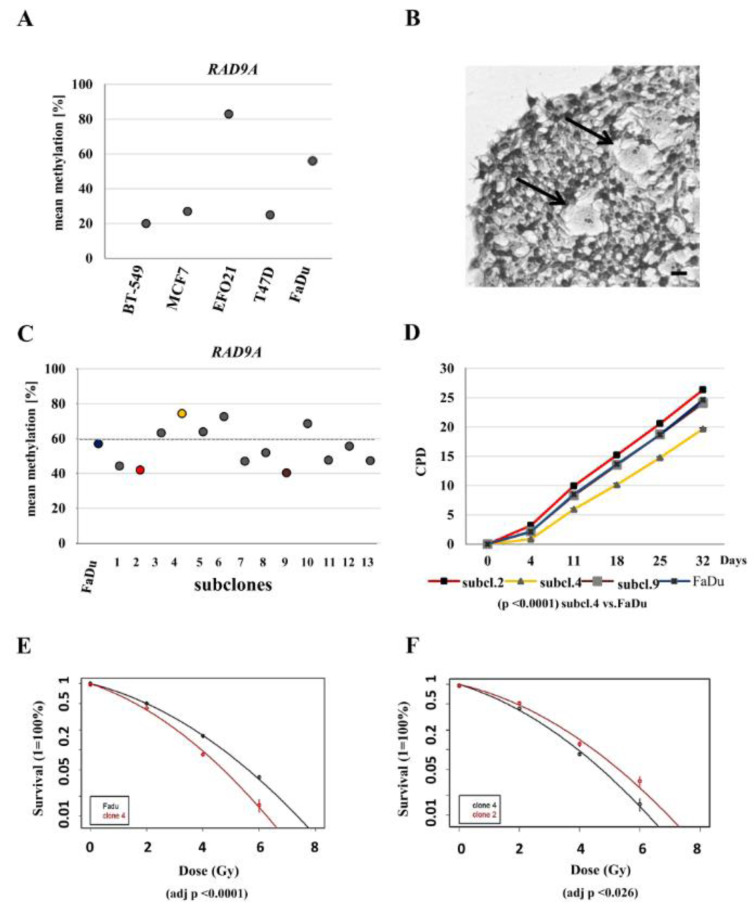
Methylation values of *RAD9A* in tumor cell lines, FaDu and FaDu subclones. (A) Mean methylation values in cell lines: BT-549, MCF7, EFO21, T47D and FaDu. (B) Crystal violet staining of an exemplary FaDu colony. Divergent cells are depicted by arrows. Scale bar, 50 µm. (C) Mean methylation of 13 FaDu subclones. The highest values were determined in subclone 4 (75 %), subclone 6 (73 %) and subclone 10 (67 %), the lowest values for subclone 9 (40 %) and subclone 2 (42 %). (D) Cumulative population doublings for subclones 2, 4, 9 and the parental FaDu cell line. Subclone 4 is delayed in growth (p-value. <0.0001). (E) Post radiation survival assay for the subclone 4 and FaDu, (F) subclones 2 and 4. Clonogenic survival was calculated as the percentage of the non-irradiated controls and is shown as means + S.D of three independent experiments. Linear quadratic fitting was performed, and results were compared using F-testing. Subclone 4 was significantly sensitive to radiation treatment in comparison to parental FaDu and subclone 2 cell line (adj p <0.0001 and adj p <0.026).

To understand the role of hypermethylation of *RAD9A* in cancer and oncogenic transformation, we further analyzed BT-549 (breast cancer), MCF7 (breast cancer), EFO-21 (ovary), T47D (breast cancer), and FaDu (squamous cell carcinoma) cell lines. The *RAD9A* methylation varied between these tumor cell lines (mean values 20-81 %, Figure 4A(Fig. 4)). The highest methylation in all three CpG's was detected in the cell line EFO-21 (CpG1-84 %, CpG2-89 %, and CpG3-76 %). The methylation values seem to be independent of the *RAD9A* copy number. The MCF7 cell line (mean methylation 24 %) has two copies of Chr.11 and one derivative der(?)t(11;1;17;19;17), in contrast to EFO-21 (mean methylation 83 %) and FaDu cell line (mean methylation 54 %) with up to three copies of chromosome 11.

If *RAD9A* methylation is relevant for tumor development it should be possible to detect divergent methylation in tumor subclones*. *Subclonal events involving gene mutations were reported by Amin et al. (2016[1]) and recently by Ben-David et al. (2018[4]) showing copy number gains and losses and consequently different drug responses in cancer (Amin et al., 2016[1]; Ben-David et al., 2018[4]). To test the hypothesis that subclonal events may be associated with methylation changes in *RAD9A* we established several subclones of the FaDu cell line. The FaDu cell line exhibits homozygous loss of function mutations in *TP53* and *CDKN2A* genes, therefore, chromosomal changes caused by the lack of proper DNA repair may occur frequently. During the cultivation of the parental FaDu cell line, subclonal events lead to divergent cells (Figure 4B(Fig. 4)).

We were able to generate thirteen stable subclonal cell lines with divergent *RAD9A* methylation (Figure 4C(Fig. 4)). Three subclones, 4, 6 and 10, exhibit high *RAD9A* methylation (74 %, 73 % and 69 % respectively) in comparison to the parental cell line (mean methylation 57 %), while others showed reduced methylation levels (e.g. subclone 2; mean 42 % and subclone 9; mean 40 %). The methylation levels of *RAD9A* remained stable during the further cultivation of all subclones. Subclones 2, 4, 6, 9 and 10 and the parental FaDu cell line were chosen for further characterization.

To elucidate if different methylation values have an impact on cell survival and damage response we performed clonogenic survival experiments. Using triplicates monolayer culture growth kinetics for the subclones 2, 4, 9 and the parental FaDu cell line cultured for 32 days (6 measuring points) revealed significantly delayed growth for subclone 4 (p <0.0001) (Figure 4D(Fig. 4)). Furthermore, clonogenic survival experiments upon irradiation performed with the subclones 2, 4 and the FaDu cell line resulted in significantly reduced survival of subclone 4 (adj p <0.0001) in comparison to the FaDu parental cell line and subclone 2 (adj p <0.026) (Figure 4 E, F(Fig. 4)). Methylation signatures for the subclones in clonogenic survival experiments matched the untreated subclone signatures (Supplementary data Figure 4).

**Figure 5:**
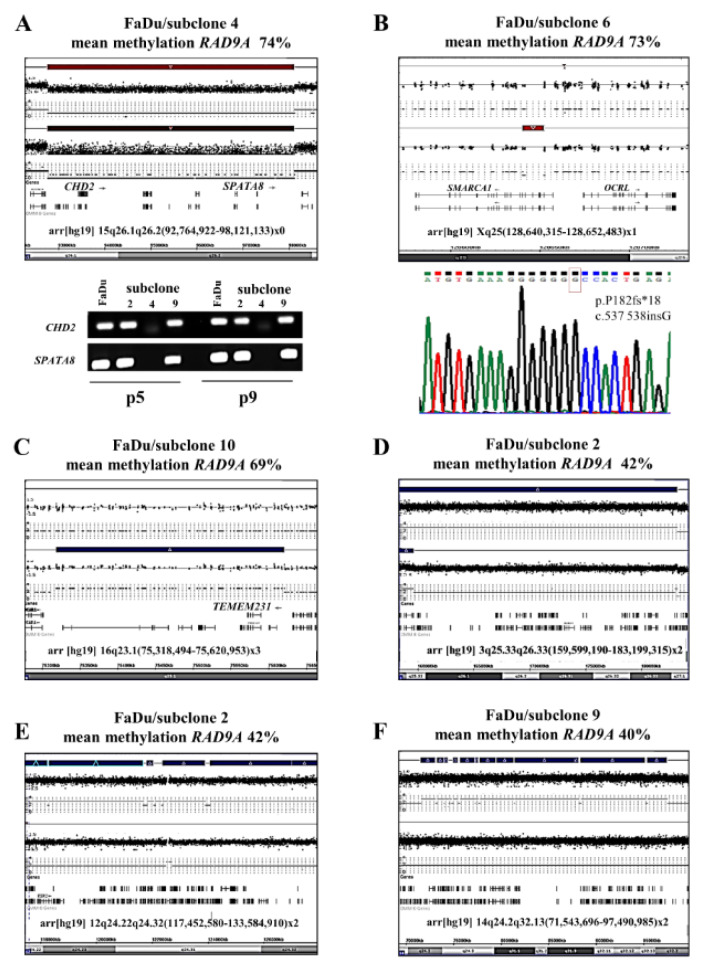
Molecular karyotyping of FaDu subclones 4, 2, 6, 9 and 10. (A) *CHD2* and *SPATA8 *are homozygously deleted in subclone 4. The upper bar mark represents the parental FaDu cell line. PCR analysis of *CDH2* and *SPATA8* genes confirmed the SNP Array result. (B) Homozygous mutation (deletion using SNP-Array and stop mutation using Sanger sequencing) is shown for *SMARCA1* in subclone 6. The analysis of A and B was conducted in two different passages (p5 and p9). (C) The subclone 10 displayed a 302 kb duplication (indicated as a blue bar) in 16q23.1(75,318,494-75,620,953). (D-F) show the restoration of duplicated areas in subclones 2 and 9. The Upper blue bar represents the duplicated chromosome section in the parental cell line FaDu. For a larger version of these graphs please see Supplementary information Figures 5A-F.

To test our hypothesis that alterations in tumor-relevant genes may be associated with changes in *RAD9A* methylation, we performed an SNP array analysis of hypermethylated (4, 6 and 10) and hypomethylated (2 and 9) FaDu subclones. We identified a homozygous deletion in 15q26.1q26.2 unique to subclone 4 and a heterozygous deletion in Xq25 in subclone 6. Downstream analysis using PCR and Sanger sequencing confirmed the homozygous deletion of the *CHD2* and *SPATA8* genes in subclone 4 and the stop codon mutation c.537-538insG, p.P182fs*18 in the remaining allele of *SMARCA1* (*SNF2L*) gene in subclone 6. Both genes harbor a helicase domain and are involved in DNA-repair and transcription regulation (Eckey et al., 2012[19]; Luijsterburg et al., 2016[53]) (Figure 5A and B(Fig. 5)).

In contrast to subclones 4, 6, and FaDu, subclone 10 displayed a 302 kb duplication in 16q23.1(75,318,494-75,620,953). The duplication encompasses 6 genes four of which are frequently deregulated in cancer (*TMEM170A, CHST6, CHST5, TMEM231*). The *TMEM231* gene is responsible for Joubert Syndrome 20; (OMIM 614970) and the *GABARAPL2 *is involved in the autophagy interaction network (Figure 5C(Fig. 5)).

The subclones 2 and 9 were hypomethylated in comparison to the parental cell line FaDu (42 % and 40 % versus 57 % mean methylation). The molecular karyotyping of these subclones revealed a restoration of former duplicated areas on chromosome 3 and 12 for subclone 2 and chromosome 14 for subclone 9 (Figure 5D-F(Fig. 5)). Chromosomal gain and gene amplification involving chromosome arm 3q are the most frequent in human tumors. The chromosomal part 3q25.33q26.33 (159,599,190-183,199,315) contains 83 genes of which 52 are involved in cancer progression. Genes within this region, like *TERC, PHC3, TNIK, MIR569, NLGN1, TBL1XR1*, *KCNMB3, ZNF639* and *USP13* are responsible for cancer development or progression when overexpressed or duplicated (Baena-Del Valle et al., 2018[3]; Chaluvally-Raghavan et al., 2014[11]; Crea et al., 2013[14]; Davidson et al., 2014[15]; Han et al., 2016[29]; Kang et al., 2009[38]; Lee et al., 2019[43]; Lin et al., 2005[49]; Xi et al., 2019[75]). The other restored part in subclone 2 is located on chromosome 12q24.22q24.32. It contains 155 Genes of which 29 are involved in cancer as a tumor suppressor, oncogene, or cancer-promoting factor. The chromosomal part restored in subclone 9 contains 114 genes, 67 of which are involved in cancer. Furthermore, the chromosome section 14q24.3 is highly amplified in 18 Head and Neck Squamous Cell Carcinoma (HNSCC) cell lines (Järvinen et al., 2008[37]) and the *TTC8* gene is responsible for the Bardet-Biedl 8 (OMIM 209900) and Retinitis pigmentosa 51 (OMIM 613464) syndrome (Supplementary data Figure 5).

### Effects of radiation and daunorubicin treatment on RAD9A methylation

**Figure 6:**
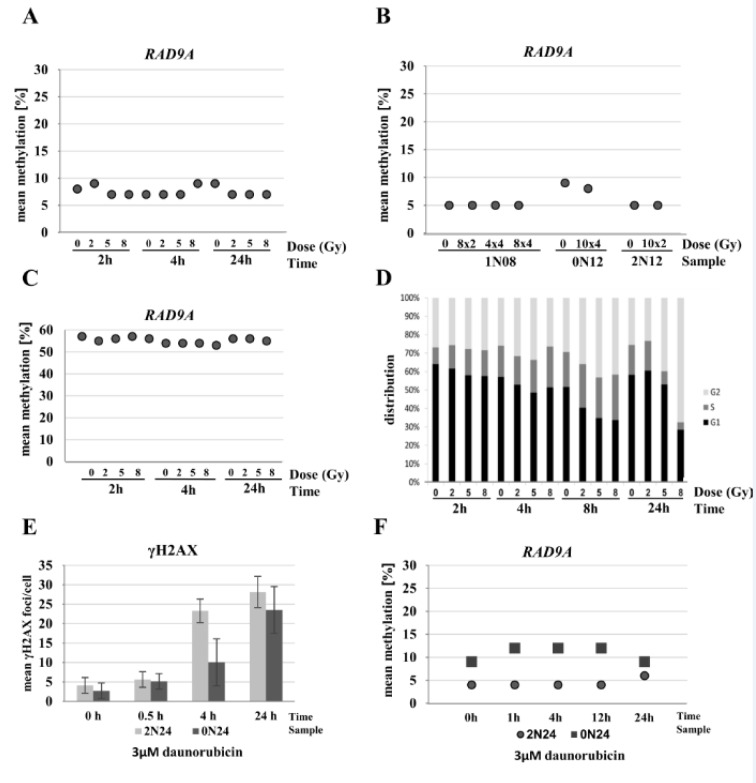
Influence of radiation and chemotherapeutics on *RAD9A* methylation. (A) Cell line 0N18 was analyzed at 15 min, 2, and 24 h after irradiation with 0 Gy, 2 Gy, 5 Gy, and 8 Gy, at each time point. Mean *RAD9A* methylation values remained virtually unchanged between 7 % and 9 %. (B) Fibroblast cell lines (0N12, 1N08, and 2N12) were irradiated in fractions of 8x 2 Gy, 4x 4 Gy, 10x 2 Gy, 8x 4 Gy, and/or 10x 4 Gy within 20 days. Mean *RAD9A* methylation remained constant at 5 % in 1N08 and 2N12, and at 8-9 % in 0N12. (C) Exponentially growing FaDu tumor cells were analyzed at 2, 4, and 24 h after irradiation with a single dose of 0 Gy, 2 Gy, 5 Gy, and 8 Gy. Mean *RAD9A* methylation varied within a narrow range between 54 % and 57 %. There was no difference between irradiated and non-irradiated cells. (D) Cell cycle analysis of FaDu cell line after treatment. The cell line was treated with increasing doses from 2 to 8 Gy. DNA was extracted at 2, 4, 8, and 24 h post-radiation. Shifts in the cell cycle phase indicate an arrest in the G2/M checkpoint. (E) Treatment of primary fibroblasts in a subconfluent state with 3 µM daunorubicin. Incorporation and toxicity of daunorubicin in corresponding samples show a time-dependent increase of γH2AX foci reflecting the DNA damage. (F) Examination of mean methylation of *RAD9A* at 0, 1, 4, 12 and 24 h post-treatment showed no changes in methylation in two independent fibroblast cell lines (2N24 and 0N24).

As most of our patients (except patient 1N20) received chemo- and radiotherapy, we designed experiments to test whether the treatment has an impact on the methylation of *RAD9A* and whether *RAD9A*-methylation can serve as a stable methylation marker. We have previously shown that DNA methylation remains stable in primary fibroblasts throughout the first cell cycle after irradiation (Maierhofer et al., 2017[54]). In contrast, significant methylation changes in >250 genes and the MAP kinase signaling pathway were associated with delayed radiation effects in single-cell clones of irradiated fibroblast (Flunkert et al., 2018[22]). To study radiation effects on the methylation of the *RAD9A *intron 2 site, the control cell line 0N18 was analyzed at 15 min, 2 and 24 h after irradiation with 0 Gy, 2 Gy, 5 Gy, and 8 Gy at each time point. The *RAD9A* mean methylation values in intron 2 remained virtually unchanged between 7 % and 9 % (Figure 6A(Fig. 6)). As *RAD9A* expression is deregulated in a variety of tumors and plays an important role in DNA repair, we additionally performed experiments with three fibroblast cultures (from patients 1N08, 0N12, and 2N12) which were irradiated in fractions of 8x 2 Gy, 4x 4 Gy, 8x 4 Gy, 10x 2 Gy, and 10x 4 Gy within 20 days. Again, *RAD9A* mean methylation remained rather constant at 5 % in 1N08 and 2N12, and at 8-9 % in 0N12 cell lines (Figure 6B(Fig. 6)). Furthermore, we performed experiments in exponentially growing FaDu tumor cells to estimate the mean methylation values of *RAD9A*. The cells were analyzed at 2, 4, and 24 h after irradiation with a single dose of 0 Gy, 2 Gy, 5 Gy, and 8 Gy. The *RAD9A* mean methylation varied within a narrow range of 54 % to 57 % and there was no difference between irradiated and non-irradiated cells (Figure 6C(Fig. 6)). The proportion of G2-phase cells (measured by flow cytometry for FaDu cells) increased with radiation dose and time after irradiation, ranging from 35 % G2-phase cells in non-irradiated cells to 65 % at 24 h after 8 Gy indicating a functional G2/M checkpoint (Figure 6D(Fig. 6)).

Although tumor therapy varied between patients, daunorubicin and doxorubicin were frequently used in the treatment regimens. As both drugs have similar properties and the cellular uptake of daunorubicin is superior to that of doxorubicin, we analyzed the influence of daunorubicin on *RAD9A* methylation. Treatment of normal fibroblasts with 3 µM daunorubicin yields a surviving cell fraction of 60 % (Przybylska et al., 2001[60]). Analysis of γH2AX as a marker for DNA double-strand breaks confirmed the incorporation and toxicity of daunorubicin in the cells (Figure 6E(Fig. 6)). Examination of the methylation signature at 0, 1, 4, 12 and 24 h post-treatment showed no significant changes in methylation in two independent fibroblast cell lines. The 2N24 cell line exhibited mean methylation values of 4-6 % and 0N24 control cell line of 9-12 % (Figure 6F(Fig. 6)) and Supplementary data Figure 6.

## Discussion

### RAD9A hypermethylation in childhood cancer

The vast majority of childhood cancers occur sporadically and cannot be explained by germline mutations in known tumor susceptibility genes (D'Orazio, 2010[18]; Sylvester et al., 2018[66]). In a previous study, we detected reduced expression levels of RAD9A in primary fibroblast cells of childhood cancer patients with a second primary tumor (2N), suggesting a function of RAD9A as a genomic caretaker (Weis et al., 2011[73]). We did not detect suspicious mutations in *RAD9A *or other cancer susceptibility genes, in our patient collective. Therefore, it seems plausible to assume that stochastic or adverse exposure events during early (intrauterine and postnatal) development may increase cancer susceptibility through epigenetic reprogramming (Marshall et al., 2014[56]; Walker and Ho et al., 2012[70]), including epimutations of *RAD9A*. We identified 5 patients (four 1N and one 2N) with ≥ 10 % mean *RAD9A* methylation in mosaic in intron 2 and using DBS analysis we could show that the mosaic epimutations represent ≥ 2 % hypermethylated alleles. Although this finding may not explain the down-regulation of RAD9A in our 2N collective, considering our experiments done in this study we propose that mosaic *RAD9A* hypermethylation is an early (either stochastic or environmentally induced) event, which may increase the probability of malignant transformation in regular body cells (predisposing factor) or/and a factor that signals the tumor progression itself (oncogenic factor) for this mainly 1N patients.

Constitutive epimutations (allele methylation errors) that arise in early development are likely to be present in a mosaic state in different tissues of an individual. Nevertheless, the risk for malignant transformations may increase with the percentage of cells with compromised genomic caretakers. Previously, we have shown that epimutations in *BRCA1* and* RAD51* can originate in single precursor cells (Böck et al., 2018[9]; Hansmann et al., 2012[30]). Furthermore, we identified a monozygotic twin pair, discordant for childhood cancer with a constitutive mosaic *BRCA1* epimutation in the twin with cancer (Galetzka et al., 2012[24]). Because studies about the involvement of mosaic epimutations in cancer are sparse (Lønning et al., 2019[50]; Hitchins, 2015[32]) it is difficult to define a threshold for constitutive epimutations in regular tissues that can be associated with tumor formation. In our experience with screening for TS epimutations in more than 800 individuals (Böck et al., 2018[9]; Hansmann et al., 2012[30]), mean methylation values of ≥ 10 % and allele methylation errors of ≥ 2-3 % (depending on the gene and assay) are outside the normal methylation variation range (depending on the tissue origin analyzed). Since all patients were analyzed using a genome-wide SNP array and no variants in *RAD9A* or neighboring genes were detected, the observed methylation changes must be caused by other mechanisms affecting gene regulation. Studies suggest that methylation may change during the lifetime (Horvath and Raj, 2018[36]). Since childhood cancer patients and controls enrolled in this study were matched, differences in *RAD9A* methylation are not be explained by age.

Although we detected 1-2 % changes in methylation values in *APC*, *CDKN2A* and *TP53* gene, it is questionable if these methylation differences are of biological significance. Epimutation rates were not significantly increased in 1N08 (*APC*), 1N15 (*CDKN2A*) and 2N12 (*TP53*) patients. When the analyzed childhood cancer patients were recruited for this study, they all had survived an initial cancer treatment (in most cases including radiation therapy) and the 2N patients had been tumor-free for several years. Although we did not find evidence for irradiation- or chemotherapy-induced RAD9A epimutations, we cannot completely exclude the possibility that the observed RAD9A hypermethylation in some patients is a consequence of cancer treatment, which may lead to mutation of important genes. Due to the dual role of RAD9A in cancer, we would assume the hypermethylation in RAD9A, seen in the five patients in mosaic, as potentially harmful, because it may activate RAD9A transcription.

### RAD9A hypermethylation in oncogenic transformation

Patient 1N20, who suffered from sporadic unilateral retinoblastoma, exhibited almost 12 % fully methylated *RAD9A* alleles in his fibroblasts, indicating that almost one-eighth of his normal cells are endowed with epimutated *RAD9A* alleles. Most retinoblastomas, which are derived from the cone photoreceptor lineage, show biallelic inactivation of the *RB1 *tumor suppressor gene, but additional (epi)genetic changes are most likely required for tumor development (Dimaras et al., 2008[17]). We did not detect an *RB1* germline mutation in our retinoblastoma patient, however, the mutational status of the tumor is not known. Instead, we detected a unique mosaic duplication in 13q14.3 including the genes *CKAP2*, *THSD1 *and* VPS36* in the patient's fibroblasts (Galetzka et al., 2020[25]). The *CKAP2* gene is a cytoskeleton-associated protein that stabilizes microtubules and plays a role in the regulation of cell division (Yoo et al., 2016[77]). Whether this gene alteration contributes to the development of sporadic retinoblastoma and/or *RAD9A *hypermethylation remains to be elucidated. The retinoblastoma of patient 1N20 was cured solely by excision of the tumor; therefore, no therapy-related changes may be expected.

The other four patients with ≥ 2 % hypermethylated alleles in *RAD9A* suffered from leukemia. Because, there is no research about the involvement of *RAD9A *hypermethylation, leukemia development and progression to date, we designed experiments with EBV, tumor cell lines, and bone marrow samples from leukemia patients to elucidate a possible mechanistic involvement.

The involvement of *RAD9A* hypermethylation in oncogenic transformation is substantiated with experiments performed with bone marrow samples from leukemia patients. We could detect a significant change in methylation values of *RAD9A* between AML and NHL patients. Moreover, we could show an evolving duplication on chr.16p13.3, which may be linked to *RAD9A* hypermethylation. One of the duplicated genes on chr.16p13.3 is *NTHL1*. Its overexpression causes genomic instability and loss of contact inhibition of cells (Limpose et al., 2018[48]). Another duplicated gene in chr.16p13.3 is *PRKD1*. High *PRKD1* mRNA levels have been associated with low overall survival in TNBC (triple-negative breast cancer) (Spasojevic et al., 2018[65]). *TNFRS12A*, also located in the 16p13.3 duplication, is responsible for poor prognosis in breast cancer, if overexpressed (Yang et al., 2018[76]). To our knowledge, this study is the first analysis of *RAD9A* methylation in leukemia and the first analysis of *RAD9A* methylation during the therapy of leukemia.

Our assumption of the possible involvement of *RAD9A* hypermethylation in unknown signaling pathways triggered by hazardous mutations compromised DNA integrity and subsequent tumor formation is further substantiated by analysis of Fanconi anemia fibroblasts. They were derived from patients with rare recessive genetic disease resulting in impaired DNA damage response and genome instability. Among those affected, the majority develop cancer, most often acute myelogenous leukemia, and 90 % develop bone marrow failure (Fiesco-Roa et al., 2019[21]). Compared to control fibroblasts we observed *RAD9A* hypermethylation in *FANCP* (homozygote deletion of *SLX4* gene) and *D1* fibroblasts (homozygous deletion of *BRCA2*).

In our experiments the EBV transformation of resting B cells to proliferating lymphoblasts induced a dramatic increase in *RAD9A* methylation and epimutation rate, indicating that epigenetic dysregulation of *RAD9A *may occur during malignant transformation early in tumorigenesis. It was reported that EBV infection causes the overexpression of *APOBEC3s* (apolipoprotein B mRNA editing catalytic polypeptide) which may increase the mutation load in the host DNA, providing further clues to the mechanisms of EBV-induced gastric carcinogenesis. This may prove to be a secondary driving force in the mutational evolution of EBV+ gastric tumors (Bobrovnitchaia et al., 2020[8]).

The methylation variation among FaDu subclones demonstrated methylation dynamics during tumor progression, which is associated with specific chromosomal rearrangements. Copy number variation is one important mechanism of cancer cells to promote the expression of genes involved in cancer progression and outcome (Chin et al., 2006[13]). In general, subclones with *RAD9A* hypermethylation showed mutations or imbalance in genes crucial for cell stability and cancer, whereas in hypomethylated clones we observed a restoration to normal copy numbers.

### RAD9A hypermethylation and gene regulation

**Figure 7:**
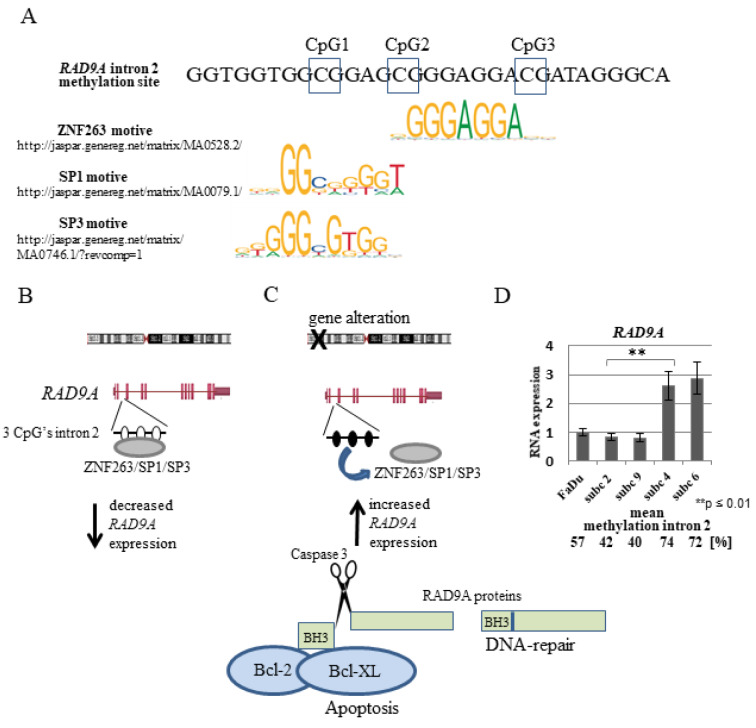
Schematic representation of the hypothetical regulation of the *RAD9A* gene expression through the methylation of *RAD9A* intron 2. (A) Putative ZNF263, SP1and SP3 binding motifs in *RAD9A* intron 2. (B, C). Demethylation CpG's in intron 2 permits the binding of ZNF263 and/or SP1, SP3 proteins decreasing *RAD9A* expression. Following genomic imbalances of tumor-relevant genes, the intron 2 site became methylated, ZNF263 and /or SP1, SP3 proteins cannot bind efficiently, in turn, the expression of *RAD9A* is enhanced. (D) RT-qPCR analysis of *RAD9A* in FaDu versus subclone 2, 9, 4, 6. The result represents the average of three independent experiments with standard deviation. In subclones with high mean methylation in *RAD9A* (74 %, 72 %) the *RAD9A* is overexpressed in comparison to the FaDu and subclones 2 and 9 (**p ≤ 0.01).

It is tempting to speculate on the existence of a signaling pathway that is activated upon deregulation of tumor-relevant genes. RAD9A has been described to be involved in DNA repair and apoptosis. When translocated to mitochondria, RAD9A binds and neutralizes the anti-apoptotic activity of BCL-2 and BCL-xL proteins, thus promoting cell death (Komatsu et al., 2000[41]; Yoshida et al., 2002[78]). How the activation, choice and fine-tuning of the pathway occurs is not known. The methylation-sensitive region in intron 2 of *RAD9A* is endowed with three regulatory elements, annotated in ORegAnno (http://www.oreganno.org/). Element OREG1137234 represents a binding site for the transcription factor ZNF263, which functions as a transcriptional repressor (Weiss et al., 2020[74]). Furthermore, it was shown that SP3, which contains an inhibitory domain to repress gene transcription, binds to the intron 2 region of the gene *in vivo *(Cheng et al., 2005[12]). It is plausible to assume that intron 2 hypermethylation interferes with SP1, SP3 and/or ZNF263 binding, and in conclusion, *RAD9A* expression is activated (Figure 7A-C(Fig. 7)). SP1 and SP3 proteins are localized exclusively in the cell nucleus (Birnbaum et al., 1995[7]; Hagen et al., 1992[28]). Both proteins contain a highly conserved DNA binding domain and two glutamine-rich regions. The glutamine-rich domains interact with the TATA box binding protein (TBP)-associated factor dTAFII110 and have a strong transcription activation function and the SP1 and SP3 (Dennig et al., 1996[16]; Gill et al., 1994[26]) when bound to the promoter region. SP3 can however also act as a transcriptional repressor due to an inhibitory domain between the second glutamine-rich domain and the zinc finger region (Luca et al., 1996[52]). It has been shown, that Sp3 binds to the intron 2 region of RAD9A *in vivo* and that in MCF-7 breast cancer cells and breast cancer tumors with increased RAD9A mRNA this binding site was hypermethylated (Cheng et al., 2005[12]). This suggests, that the unmethylated intron 2 SP1/SP3 binding site is bound by SP3, reducing the transcription of RAD9A, whereas hypermethylation inhibits SP3 binding and leading to increased RAD9A expression.

## Conclusion

According to the Cancer Genome Atlas/TCGA (https://cancergenome.nih.gov/), *RAD9A* is overexpressed in a wide variety of tumors including leukemia and solid tumors, supporting its oncogenic function but the relationship between tumor type, survival and *RAD9A* hypermethylation demands studying larger patients cohorts. Stable methylation markers could specify more precise diagnosis and therapy outcome, but very few are on their way to clinical application. We propose that *RAD9A* methylation may serve as a marker, for the cell fate and possible therapy outcomes, as suggested for prostate cancer (Lieberman et al., 2018[46]). We are aware that the patients presented here, were selected based on survival criteria. Therefore, larger prospective studies in various cancer types and tissues are needed to generally correlate *RAD9A* epimutations with cancer incidence and therapy outcomes. 

## Notes

Danuta Galetzka, Julia Böck and Lukas Wagner contributed equally as first author.

## Declaration

### Acknowledgments

First of all, we would like to thank the patients for participating in this study. Our clinical staff also deserves special thanks. We also thank Ursula Disque-Kaiser, Britta Weber, Danuta Wieczorek, Martina Mihm, Brigitte Schuh and Martina Hermanns for technical support. We kindly thank Prof. Detlev Schindler for the donation of Fanconi Fibroblasts. We are grateful to Benjamin Irmscher for providing the daunorubicin experiments. Special thanks to Heather Chorzempa for the proofreading of this manuscript.

### Availability of data and materials

The datasets generated and analyzed during the current study are not publicly available due to ethics and data protection reasons but are available from the corresponding author on reasonable request.

### Conflict of interest

The authors declare no conflict of interest

### Funding

This study was supported by the BMBF German ministry for education and science [02NUK016A, 02NUK042A, 2NUK042B and 2NUK042C] and has received funding from the Brigitte und Dr. Konstanze Wegener-Stiftung (Project #70).

### Authors' contributions

**DG, JB **and **LW **contributed equally to this work, and should be considered co-first authors, **DG** is the guarantor of this work and, as such, had full access to all of the data in the study and takes responsibility for the integrity of the data and the accuracy of the data analysis. **MD **analyzed the bNGS data. **DG, OS, ML**, **JM,** prepared cell culture radiation and pyrosequencing experiments.** AP** performed the statistical analysis of the pyro sequence data, the colony formation assay and the growth kinetics. **MR, LW** and **WB** prepared the tumor cell lines and bone marrow samples. **MLi** and **DG** conducted the SNP array molecular karyotyping. **HR** helped with the analysis of pyro sequence data. **JB** and **OS** prepared the bisulfite sequence bNGS and provided the data interpretation. **CS, MM**, **PSZ** organized the patient's recruitment. **DG, TH, DP** and **HS** conceived and designed this project and wrote the paper. All authors revised the manuscript critically for important intellectual content and approved the final version and agreed to be accountable for all aspects of the work in ensuring that questions related to the accuracy or integrity of any part of the work are appropriately investigated and resolved.

## Supplementary Material

Supplementary data

Supplementary information

## Figures and Tables

**Table 1:**
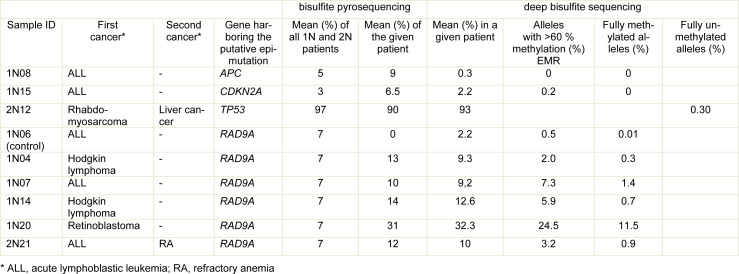
Results of methylation analysis (by bisulfite pyrosequencing and deep bisulfite sequencing) of patients with suspected epimutations

## References

[1] Amin NA, Seymour E, Saiya-Cork K, Parkin B, Shedden K, Malek SN (2016). A quantitative analysis of subclonal and clonal gene mutations before and after therapy in chronic lymphocytic leukemia. Clin Cancer Res.

[2] Aubrey BJ, Strasser A, Kelly GL (2016). Tumor-Suppressor Functions of the TP53 Pathway. Cold Spring Harb Perspect Med.

[3] Baena-Del Valle JA, Zheng Q, Esopi DM, Rubenstein M, Hubbard GK, Moncaliano MC (2018). MYC drives overexpression of telomerase RNA (hTR/TERC) in prostate cancer. J Pathol.

[4] Ben-David U, Siranosian B, Ha G, Tang H, Oren Y, Hinohara K (2018). Genetic and transcriptional evolution alters cancer cell line drug response. Nature.

[5] Benjamini Y, Hochberg Y (1995). Controlling the false discovery rate: a practical and powerful ap-proach to multiple testing. J R Stat Soc Series B.

[6] Bennett-Baker PE, Wilkowski J, Burke DT (2003). Age-associated activation of epigenetically repressed genes in the mouse. Genetics.

[7] Birnbaum MJ, van Wijnen AJ, Odgren PR, Last TJ, Suske G, Stein GS (1995). Sp1 trans-activation of cell cycle regulated promoters is selectively repressed by Sp3. Biochemistry.

[8] Bobrovnitchaia I, Valieris R, Drummond RD, Lima JP, Freitas HC, Bartelli TF (2020). APOBEC-mediated DNA alterations: A possible new mechanism of carcinogenesis in EBV-positive gastric cancer. Int J Cancer.

[9] Böck J, Appenzeller S, Haertle L, Schneider T, Gehrig A, Schröder J (2018). Single CpG hypermethylation, allele methylation errors, and decreased expression of multiple tumor suppressor genes in normal body cells of mutation-negative early-onset and high-risk breast cancer patients. Int J Cancer.

[10] Broustas CG, Zhu A, Lieberman HB (2012). Rad9 protein contributes to prostate tumor progression by promoting cell migration and anoikis resistance. J Biol Chem.

[11] Chaluvally-Raghavan P, Zhang F, Pradeep S, Hamilton MP, Zhao X, Rupaimoole R (2014). Copy number gain of hsa-miR-569 at 3q26.2 leads to loss of TP53INP1 and aggressiveness of epithelial cancers. Cancer Cell.

[12] Cheng CK, Chow LWC, Loo WTY, Chan TK, Chan V (2005). The cell cycle checkpoint gene Rad9 is a novel oncogene activated by 11q13 amplification and DNA methylation in breast cancer. Cancer Res.

[13] Chin K, DeVries S, Fridlyand J, Spellman PT, Roydasgupta R, Kuo W-L (2006). Genomic and transcriptional aberrations linked to breast cancer pathophysiologies. Cancer Cell.

[14] Crea F, Sun L, Pikor L, Frumento P, Lam WL, Helgason CD (2013). Mutational analysis of Polycomb genes in solid tumours identifies PHC3 amplification as a possible cancer-driving genetic alteration. Br J Cancer.

[15] Davidson B, Abeler VM, Førsund M, Holth A, Yang Y, Kobayashi Y (2014). Gene expression signatures of primary and metastatic uterine leiomyosarcoma. Hum Pathol.

[16] Dennig J, Beato M, Suske G (1996). An inhibitor domain in Sp3 regulates its glutamine-rich activation domains. EMBO J.

[17] Dimaras H, Khetan V, Halliday W, Orlic M, Prigoda NL, Piovesan B (2008). Loss of RB1 induces non-proliferative retinoma: increasing genomic instability correlates with progression to retinoblastoma. Hum Mol Genet.

[18] D'Orazio JA (2010). Inherited cancer syndromes in children and young adults. J Pediatr Hematol Oncol.

[19] Eckey M, Kuphal S, Straub T, Rümmele P, Kremmer E, Bosserhoff AK (2012). Nucleosome remodeler SNF2L suppresses cell proliferation and migration and attenuates Wnt signaling. Mol Cell Biol.

[20] Etzold A, Galetzka D, Weis E, Bartsch O, Haaf T, Spix C (2016). CAF-like state in primary skin fibroblasts with constitutional BRCA1 epimutation sheds new light on tumor suppressor deficiency-related changes in healthy tissue. Epigenetics.

[21] Fiesco-Roa MO, Giri N, McReynolds LJ, Best AF, Alter BP (2019). Genotype-phenotype associations in Fanconi anemia: A literature review. Blood Rev.

[22] Flunkert J, Maierhofer A, Dittrich M, Müller T, Horvath S, Nanda I (2018). Genetic and epigenetic changes in clonal descendants of irradiated human fibroblasts. Exp Cell Res.

[23] Gale KB, Ford AM, Repp R, Borkhardt A, Keller C, Eden OB (1997). Backtracking leukemia to birth: Identification of clonotypic gene fusion sequences in neonatal blood spots. Proc Natl Acad Sci U S A.

[24] Galetzka D, Hansmann T, El Hajj N, Weis E, Irmscher B, Ludwig M (2012). Monozygotic twins discordant for constitutive BRCA1 promoter methylation, childhood cancer and secondary cancer. Epigenetics.

[25] Galetzka D, Müller T, Dittrich M, Endres M, Kartal N, Sinizyn O (2020). Molecular karyotyping and gene expression analysis in childhood cancer patients. J Mol Med.

[26] Gill G, Pascal E, Tseng ZH, Tjian R (1994). A glutamine-rich hydrophobic patch in transcription factor Sp1 contacts the dTAFII110 component of the Drosophila TFIID complex and mediates transcriptional activation. Proc Natl Acad Sci U S A.

[27] Goel A, Nguyen T-P, Leung H-CE, Nagasaka T, Rhees J, Hotchkiss E (2011). De novo constitutional MLH1 epimutations confer early-onset colorectal cancer in two new sporadic Lynch syndrome cases, with derivation of the epimutation on the paternal allele in one. Int J Cancer.

[28] Hagen G, Müller S, Beato M, Suske G (1992). Cloning by recognition site screening of two novel GT box binding proteins: a family of Sp1 related genes. Nucleic Acids Res.

[29] Han C, Yang L, Choi HH, Baddour J, Achreja A, Liu Y (2016). Amplification of USP13 drives ovarian cancer metabolism. Nat Commun.

[30] Hansmann T, Pliushch G, Leubner M, Kroll P, Endt D, Gehrig A (2012). Constitutive promoter methylation of BRCA1 and RAD51C in patients with familial ovarian cancer and early-onset sporadic breast cancer. Hum Mol Genet.

[31] Hernandez-Vargas H, Gruffat H, Cros MP, Diederichs A, Sirand C, Vargas-Ayala RC (2017). Viral driven epigenetic events alter the expression of cancer-related genes in Epstein-Barr-virus naturally infected Burkitt lymphoma cell lines. Sci Rep.

[32] Hitchins MP (2015). Constitutional epimutation as a mechanism for cancer causality and heritability?. Nat Rev Cancer.

[33] Hitchins MP (2016). Finding the needle in a haystack: identification of cases of Lynch syndrome with MLH1 epimutation. Fam Cancer.

[34] Hopkins KM, Auerbach W, Wang XY, Hande MP, Hang H, Wolgemuth DJ (2004). Deletion of mouse rad9 causes abnormal cellular responses to DNA damage, genomic instability, and embryonic lethality. Mol Cell Biol.

[35] Horsthemke B (2006). Epimutations in human disease. Curr Top Microbiol Immunol.

[36] Horvath S, Raj K (2018). DNA methylation-based biomarkers and the epigenetic clock theory of ageing. Nat Rev Genet.

[37] Järvinen A-K, Autio R, Kilpinen S, Saarela M, Leivo I, Grénman R (2008). High-resolution copy number and gene expression microarray analyses of head and neck squamous cell carcinoma cell lines of tongue and larynx. Genes Chromosom. Cancer.

[38] Kang JU, Koo SH, Kwon KC, Park JW, Kim JM (2009). Identification of novel candidate target genes, including EPHB3, MASP1 and SST at 3q26.2-q29 in squamous cell carcinoma of the lung. BMC Cancer.

[39] Karakosta A, Golias C, Charalabopoulos A, Peschos D, Batistatou A, Charalabopoulos K (2005). Genetic models of human cancer as a multistep process. Paradigm models of colorectal cancer, breast cancer, and chronic myelogenous and acute lymphoblastic leukaemia. J Exp Clin Cancer Res.

[40] Knudson AG (1996). Hereditary cancer: two hits revisited. J Cancer Res Clin Oncol.

[41] Komatsu K, Miyashita T, Hang H, Hopkins KM, Zheng W, Cuddeback S (2000). Human homologue of S. pombe Rad9 interacts with BCL-2/BCL-xL and promotes apoptosis. Nat Cell Biol.

[42] Kuhlen M, Taeubner J, Brozou T, Wieczorek D, Siebert R, Borkhardt A (2019). Family-based germline sequencing in children with cancer. Oncogene.

[43] Lee RS, Zhang L, Berger A, Lawrence MG, Song J, Niranjan B (2019). Characterization of the ERG-regulated Kinome in Prostate Cancer Identifies TNIK as a Potential Therapeutic Target. Neoplasia.

[44] Lieberman HB, Bernstock JD, Broustas CG, Hopkins KM, Leloup C, Zhu A (2011). The role of RAD9 in tumorigenesis. J Mol Cell Biol.

[45] Lieberman HB, Panigrahi SK, Hopkins KM, Wang L, Broustas CG (2017). p53 and RAD9, the DNA damage response, and regulation of transcription networks. Radiat Res.

[46] Lieberman HB, Rai AJ, Friedman RA, Hopkins KM, Broustas CG (2018). Prostate cancer: unmet clinical needs and RAD9 as a candidate biomarker for patient management. Transl Cancer Res.

[47] Ligtenberg MJL, Kuiper RP, Chan TL, Goossens M, Hebeda KM, Voorendt M (2009). Heritable somatic methylation and inactivation of MSH2 in families with Lynch syndrome due to deletion of the 3' exons of TACSTD1. Nat Genet.

[48] Limpose KL, Trego KS, Li Z, Leung SW, Sarker AH, Shah JA (2018). Overexpression of the base excision repair NTHL1 glycosylase causes genomic instability and early cellular hallmarks of cancer. Nucleic Acids Res.

[49] Lin S-C, Liu C-J, Ko S-Y, Chang H-C, Liu T-Y, Chang K-W (2005). Copy number amplification of 3q26-27 oncogenes in microdissected oral squamous cell carcinoma and oral brushed samples from areca chewers. J Pathol.

[50] Lønning PE, Eikesdal HP, Løes IM, Knappskog S (2019). Constitutional Mosaic Epimutations - a hidden cause of cancer?. Cell Stress.

[51] Lopez-Lazaro M (2018). The stem cell division theory of cancer. Crit Rev Oncol Hematol.

[52] Luca P de, Majello B, Lania L (1996). Sp3 represses transcription when tethered to promoter DNA or targeted to promoter proximal RNA. J Biol Chem.

[53] Luijsterburg MS, Krijger I de, Wiegant WW, Shah RG, Smeenk G, Groot AJL de (2016). PARP1 Links CHD2-Mediated Chromatin Expansion and H3.3 Deposition to DNA Repair by Non-homologous End-Joining. Mol Cell.

[54] Maierhofer A, Flunkert J, Dittrich M, Müller T, Schindler D, Nanda I (2017). Analysis of global DNA methylation changes in primary human fibroblasts in the early phase following X-ray irradiation. PLoS ONE.

[55] Marron, M (2017). Cancer in childhood and molecular epidemiology - The KIKME case-control study [abstract]. Cancer Research.

[56] Marshall GM, Carter DR, Cheung BB, Liu T, Mateos MK, Meyerowitz JG (2014). The prenatal origins of cancer. Nat Rev Cancer.

[57] McHale CM, Wiemels JL, Zhang L, Ma X, Buffler PA, Feusner J (2003). Prenatal origin of childhood acute myeloid leukemias harboring chromosomal rearrangements t(15;17) and inv(16). Blood.

[58] Menegakis A, Yaromina A, Eicheler W, Dörfler A, Beuthien-Baumann B, Thames HD (2009). Prediction of clonogenic cell survival curves based on the number of residual DNA double strand breaks measured by gammaH2AX staining. Int J Radiat Biol.

[59] Neitzel H (1986). A routine method for the establishment of permanent growing lymphoblastoid cell lines. Hum Genet.

[60] Przybylska M, Koceva-Chyla A, Rózga B, Józwiak Z (2001). Cytotoxicity of daunorubicin in trisomic (+21) human fibroblasts: relation to drug uptake and cell membrane fluidity. Cell Biol Int.

[61] R Core Team (2021). R: A language and environment for statistical computing. https://www.R-project.org/.

[62] Rahmann S, Beygo J, Kanber D, Martin M, Horsthemke B, Buiting K (2013). Amplikyzer: Automated methylation analysis of amplicons from bisulfite flowgram sequencing. https://peerj.com/preprints/122/.

[63] Raval A, Tanner SM, Byrd JC, Angerman EB, Perko JD, Chen S-S (2007). Downregulation of death-associated protein kinase 1 (DAPK1) in chronic lymphocytic leukemia. Cell.

[64] Ruijter JM, Lorenz P, Tuomi JM, Hecker M, van den Hoff MJB (2014). Fluorescent-increase kinetics of different fluorescent reporters used for qPCR depend on monitoring chemistry, targeted sequence, type of DNA input and PCR efficiency. Microchim Acta.

[65] Spasojevic C, Marangoni E, Vacher S, Assayag F, Meseure D, Château-Joubert S (2018). PKD1 is a potential biomarker and therapeutic target in triple-negative breast cancer. Oncotarget.

[66] Sylvester DE, Chen Y, Jamieson RV, Dalla-Pozza L, Byrne JA (2018). Investigation of clinically relevant germline variants detected by next-generation sequencing in patients with childhood cancer: a review of the literature. J Med Genet.

[67] Tomasetti C, Li L, Vogelstein B (2017). Stem cell divisions, somatic mutations, cancer etiology, and cancer prevention. Science.

[68] Tsai F-L, Kai M (2014). The checkpoint clamp protein Rad9 facilitates DNA-end resection and prevents alternative non-homologous end joining. Cell Cycle.

[69] Vockerodt M, Yap L-F, Shannon-Lowe C, Curley H, Wei W, Vrzalikova K (2015). The Epstein-Barr virus and the pathogenesis of lymphoma. J Pathol.

[70] Walker CL, Ho S-m (2012). Developmental reprogramming of cancer susceptibility. Nat Rev Cancer.

[71] Weber M, Hellmann I, Stadler MB, Ramos L, Pääbo S, Rebhan M (2007). Distribution, silencing potential and evolutionary impact of promoter DNA methylation in the human genome. Nat Genet.

[72] Weis E, Galetzka D, Herlyn H, Schneider E, Haaf T (2008). Humans and chimpanzees differ in their cellular response to DNA damage and non-coding sequence elements of DNA repair-associated genes. Cytogenet Genome Res.

[73] Weis E, Schoen H, Victor A, Spix C, Ludwig M, Schneider-Raetzke B (2011). Reduced mRNA and protein expression of the genomic caretaker RAD9A in primary fibroblasts of individuals with childhood and independent second cancer. PLoS ONE.

[74] Weiss RJ, Spahn PN, Toledo AG, Chiang AWT, Kellman BP, Li J (2020). ZNF263 is a transcriptional regulator of heparin and heparan sulfate biosynthesis. Proc Natl Acad Sci U S A.

[75] Xi X, Wu Q, Bao Y, Lin M, Zhong X, Dai X (2019). Overexpression of TBL1XR1 confers tumorigenic capability and promotes recurrence of osteosarcoma. Eur J Pharmacol.

[76] Yang J, Min K-W, Kim D-H, Son BK, Moon KM, Wi YC (2018). High TNFRSF12A level associated with MMP-9 overexpression is linked to poor prognosis in breast cancer: Gene set enrichment analysis and validation in large-scale cohorts. PLoS ONE.

[77] Yoo BH, Park C-H, Kim H-J, Kang D-S, Bae C-D (2016). CKAP2 is necessary to ensure the faithful spindle bipolarity in a dividing diploid hepatocyte. Biochem Biophys Res Commun.

[78] Yoshida K, Komatsu K, Wang H-G, Kufe D (2002). c-Abl tyrosine kinase regulates the human Rad9 checkpoint protein in response to DNA damage. Mol Cell Biol.

[79] Zhu A, Zhang CX, Lieberman HB (2008). Rad9 has a functional role in human prostate carcinogenesis. Cancer Res.

[80] Zhu A, Zhou H, Leloup C, Marino SA, Geard CR, Hei TK (2005). Differential impact of mouse Rad9 deletion on ionizing radiation-induced bystander effects. Radiat Res.

