# Supplementary material for: Hypermethylation of RAD9A intron 2 in childhood cancer patients, leukemia and tumor cell lines suggest a role for oncogenic transformation
Source: EXCLI J. 2022 Jan 7;21:117–43. doi: 10.17179/excli2021-4482 (PMC8859646; doi:10.17179/excli2021-4482)
Supplement: Supplementary information [file EXCLI-21-117-s-002.pdf]

EXCLI Journal 2022;21:117-143 – ISSN 1611-2156
Received: November 02, 2021, accepted: December 15, 2021, published: January 07, 2022

Supplementary information to:

Original article:

HYPERMETHYLATION OF *RAD9A* INTRON 2 IN CHILDHOOD CANCER PATIENTS, LEUKEMIA AND TUMOR CELL LINES SUGGEST A ROLE FOR ONCOGENIC TRANSFORMATION

Danuta Galetzka[1¶], Julia Böck[2,15¶], Lukas Wagner[14¶], Marcus Dittrich[3], Olesja Sinizyn[1], Marco Ludwig[4], Heidi Rossmann[5], Claudia Spix[6], Markus Radsak[7], Peter Scholz-Kreisel[8], Johanna Mirsch[9], Matthias Linke[10], Walburgis Brenner[11], Manuela Marron[12], Alicia Poplawski[13], Thomas Haaf[2], Heinz Schmidberger[1], Dirk Prawitt[14*]

1 Department of Radiation Oncology and Radiation Therapy, University Medical Centre, Mainz, Germany
2 Institute of Human Genetics, Julius Maximilians University, Würzburg, Germany
3 Bioinformatics Department, Julius Maximilians University, Würzburg, Germany
4 DRK Medical Centre, Alzey, Germany
5 Institute of Clinical Chemistry and Laboratory Medicine, University Medical Centre, Mainz, Germany
6 Division of Childhood Cancer Epidemiology, Institute of Medical Biostatistics, Epidemiology and Informatics, University Medical Centre, Mainz, Germany
7 Department of Hematology, University Medical Centre, Mainz, Germany
8 Federal Office of Radiation, Neuherberg, Germany
9 Radiation Biology and DNA Repair, Technical University of Darmstadt, Germany
10 Institute of Human Genetics, University Medical Centre, Mainz, Germany
11 Department of Obstetrics and Women's Health, University Medical Centre, Mainz, Germany
12 Leibniz Institute for Prevention Research and Epidemiology – BIPS, Bremen, Germany
13 Institute of Medical Biostatistics, Epidemiology and Informatics, University Medical Centre, Mainz, Germany
14 Center for Pediatrics and Adolescent Medicine, University Medical Centre, Mainz, Germany
15 Institute of Pathology, Julius Maximilians University, Würzburg, Germany

* **Corresponding author:** Dirk Prawitt, Center for Pediatrics and Adolescent Medicine, University Medical Centre, Langenbeckstraße 1, Uni-Klinik Geb. 109, 55131 Mainz, Germany, E-mail: dprawitt@uni-mainz.de

¶ These authors contributed equally to this work.

https://dx.doi.org/10.17179/excli2021-4482

This is an Open Access article distributed under the terms of the Creative Commons Attribution License (http://creativecommons.org/licenses/by/4.0/).

EXCLI Journal 2022;21:117-143 – ISSN 1611-2156
Received: November 02, 2021, accepted: December 15, 2021, published: January 07, 2022

E

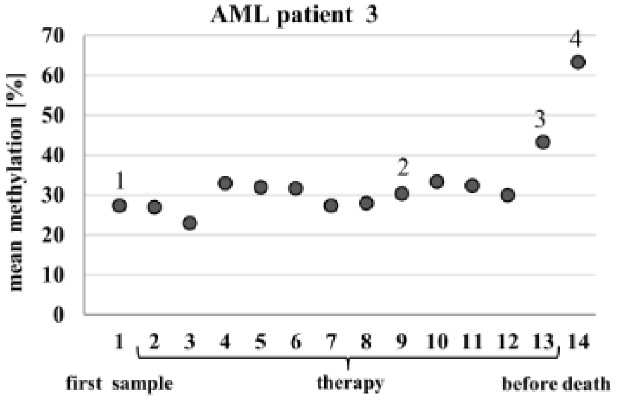

F

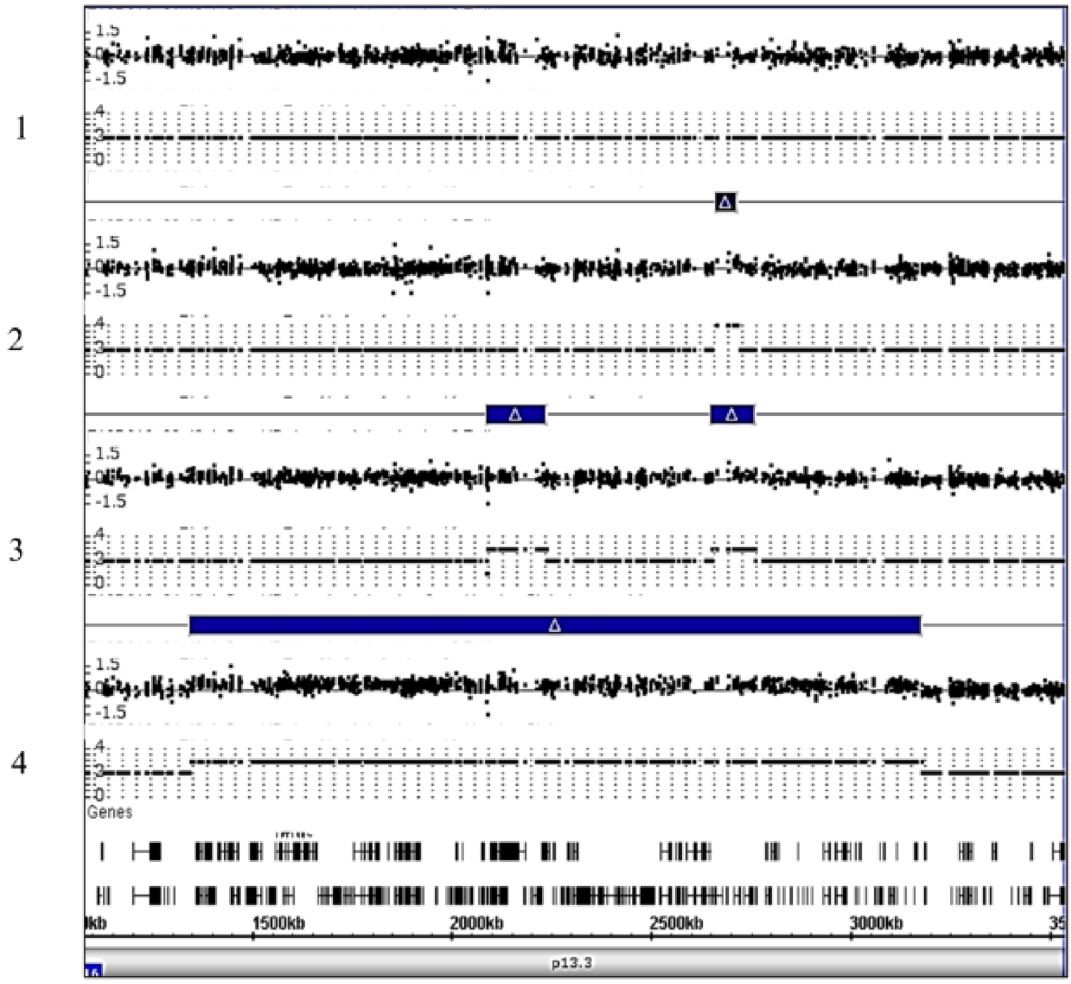

arr[hg19] 16p13.3(1,345,222-3,178,084)x3

Supplementary Figure 2E, F

EXCLI Journal 2022;21:117-143 – ISSN 1611-2156
Received: November 02, 2021, accepted: December 15, 2021, published: January 07, 2022

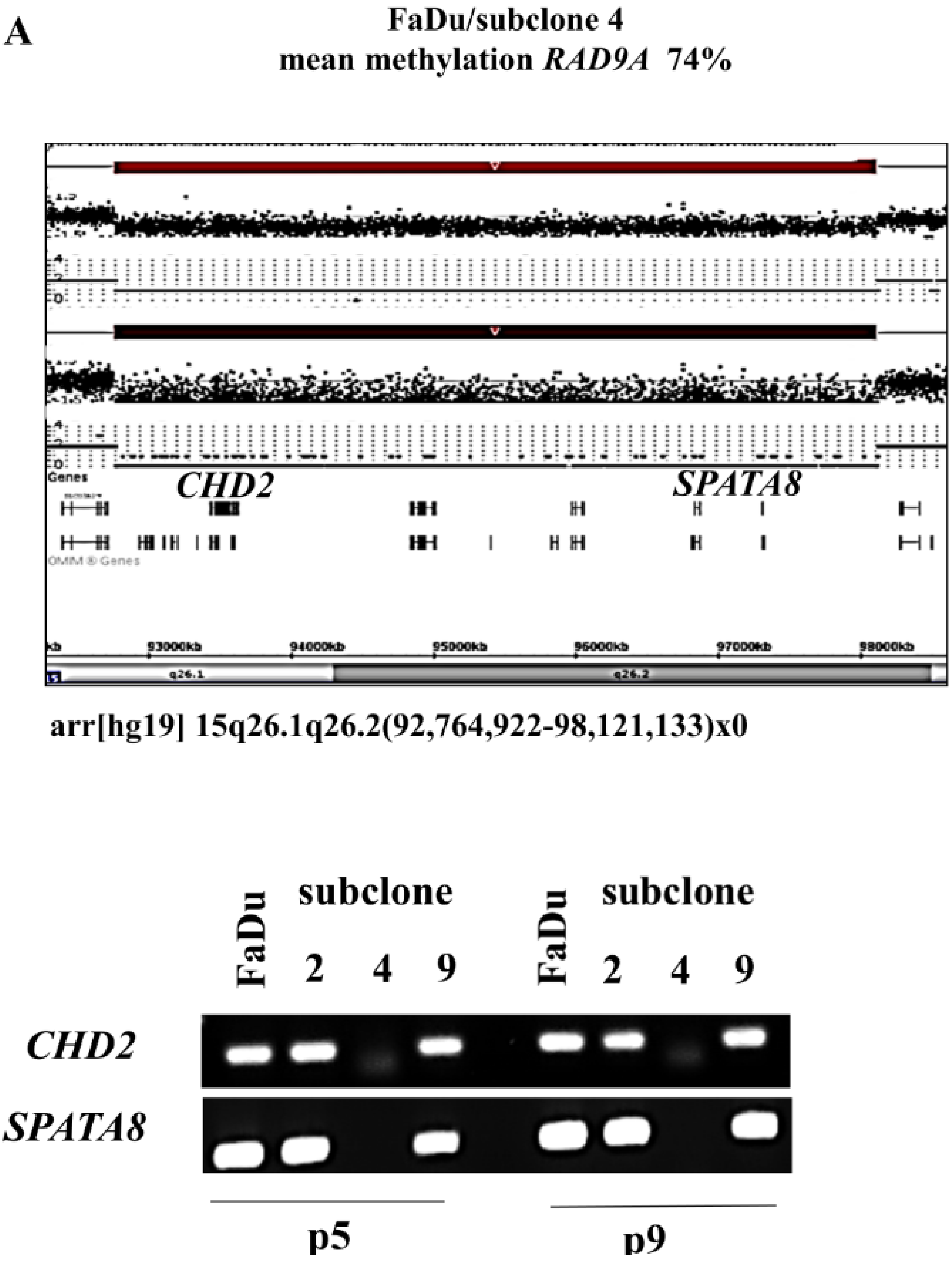

Supplementary Figure 5A: Molecular karyotyping of FaDu subclones 4, 2, 6, 9 and 10. (A) *CHD2* and *SPATA8* are homozygously deleted in subclone 4. The upper bar mark represents the parental FaDu cell line. PCR analysis of *CDH2* and *SPATA8* genes confirmed the SNP Array result.

EXCLI Journal 2022;21:117-143 – ISSN 1611-2156
Received: November 02, 2021, accepted: December 15, 2021, published: January 07, 2022

FaDu/subclone 6
mean methylation *RAD9A* 73%

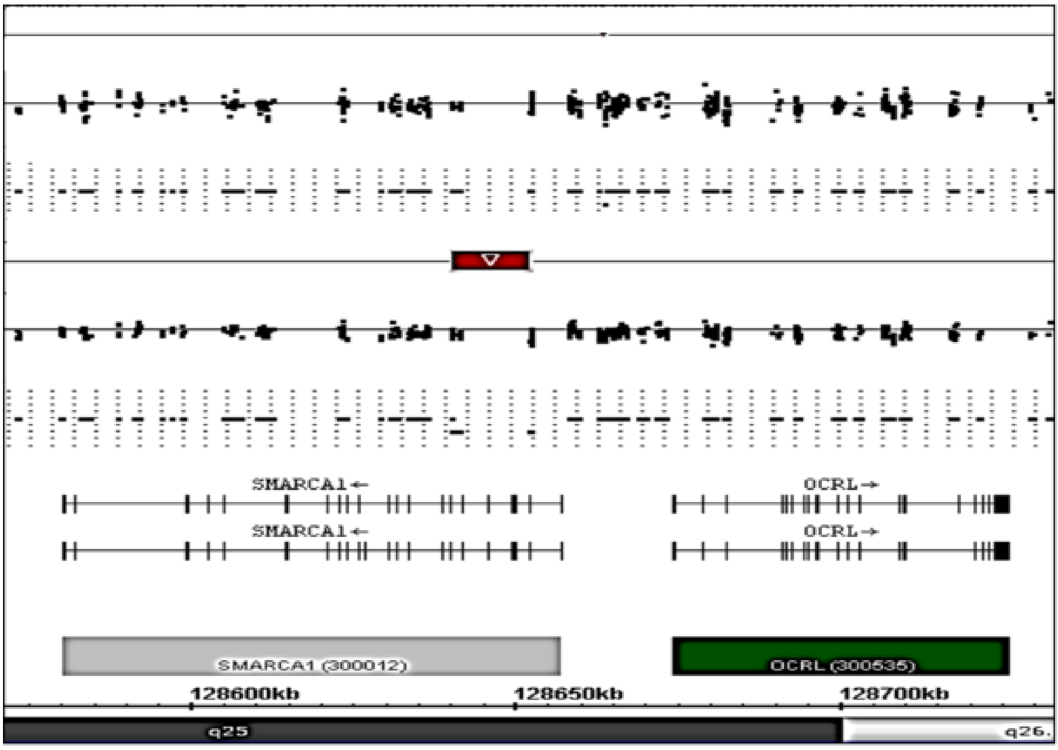

arr[hg19] Xq25(128,640,315-128,652,483)x1

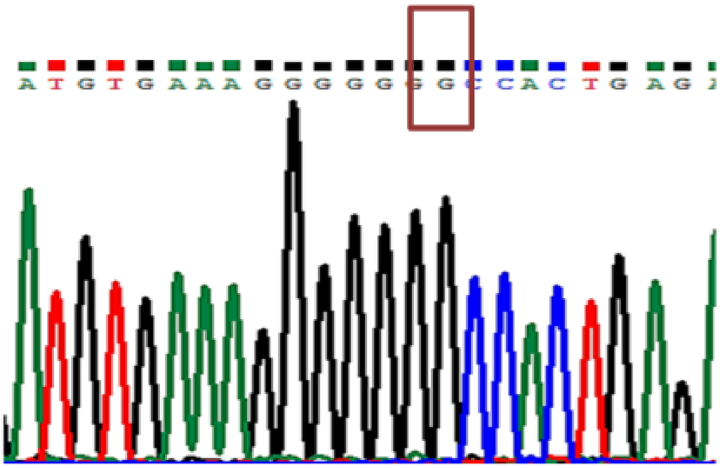

p.P182fs*18
c.537 538insG

Supplementary Figure 5B: Homozygous mutation (deletion using SNP-Array and stop mutation using Sanger sequencing) is shown for *SMARCA1* in subclone 6. The analysis of **A** and **B** was conducted in two different passages (p5 and p9).

EXCLI Journal 2022;21:117-143 – ISSN 1611-2156
Received: November 02, 2021, accepted: December 15, 2021, published: January 07, 2022

FaDu/subclone 10
mean methylation *RAD9A* 69%

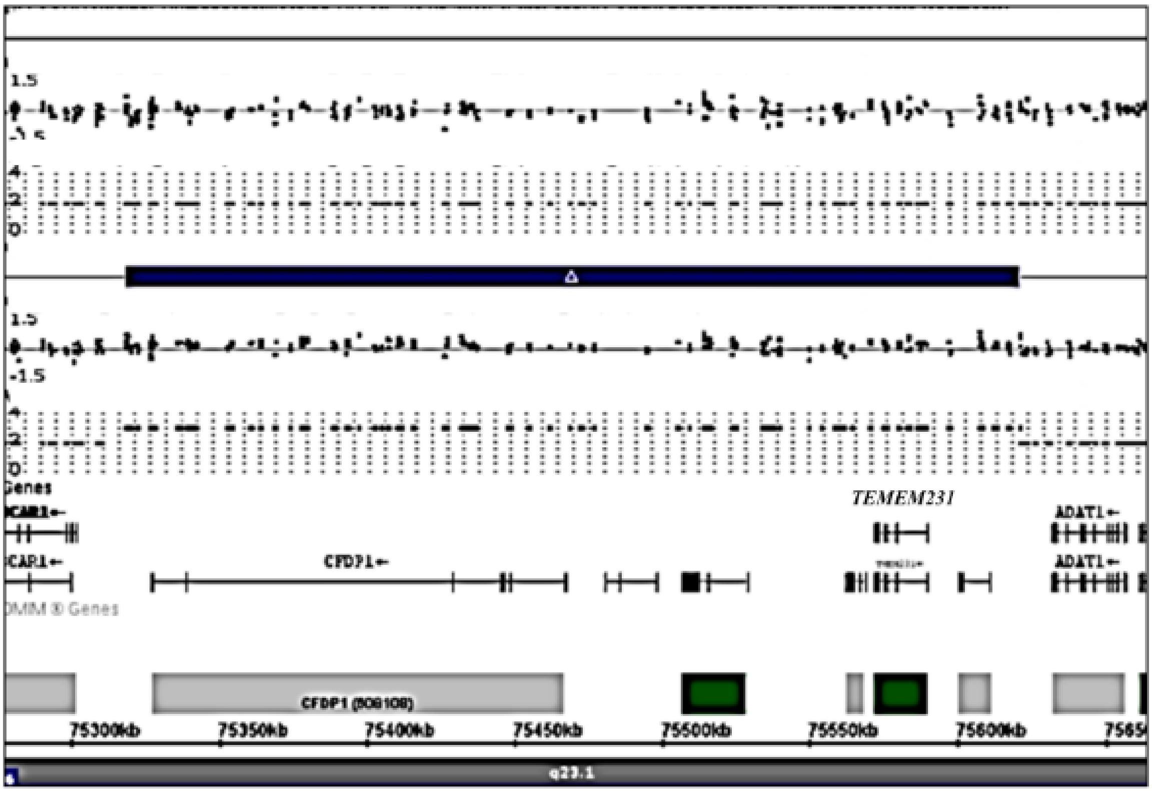

arr [hg19] 16q23.1(75,318,494-75,620,953)x3

Supplementary Figure 5C: The subclone 10 displayed a 302 kb duplication (indicated as a blue bar) in 16q23.1(75,318,494-75,620,953).

EXCLI Journal 2022;21:117-143 – ISSN 1611-2156
Received: November 02, 2021, accepted: December 15, 2021, published: January 07, 2022

FaDu/subclone 2
mean methylation *RAD9A* 42%

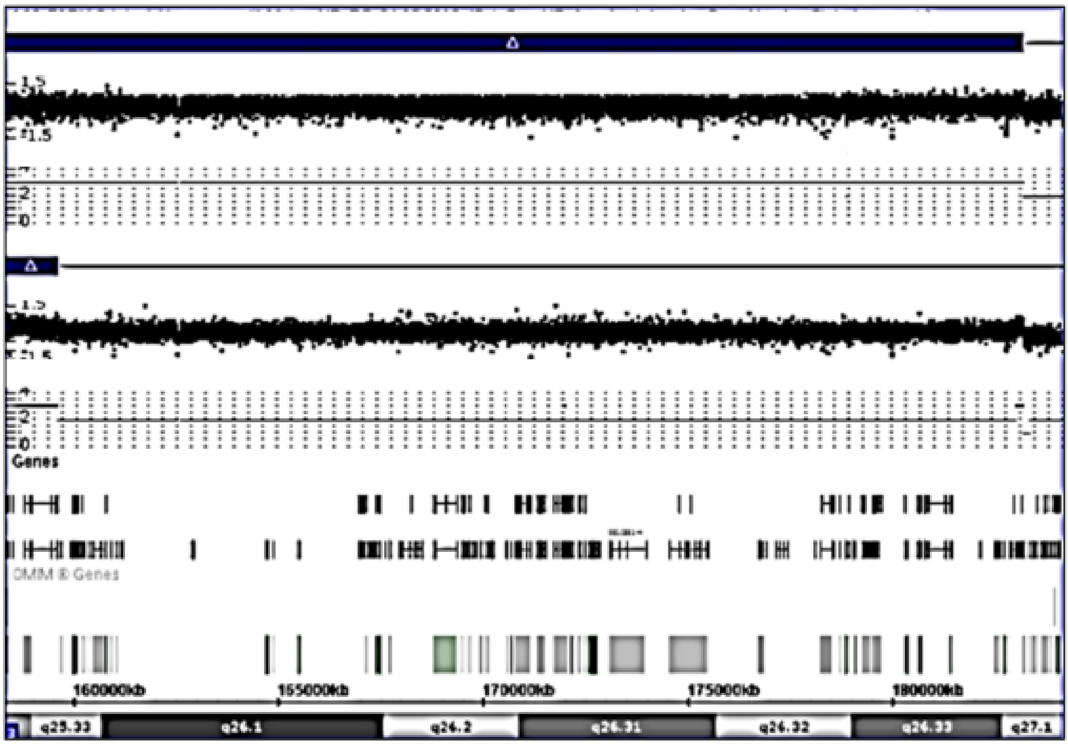

arr [hg19] 3q25.33q26.33(159,599,190-183,199,315)x2

Supplementary Figure 5D-F show the restoration of duplicated areas in subclones 2 and 9. The upper blue bar represents the duplicated chromosome section in the parental cell line FaDu.

EXCLI Journal 2022;21:117-143 – ISSN 1611-2156
Received: November 02, 2021, accepted: December 15, 2021, published: January 07, 2022

FaDu/subclone 2
mean methylation *RAD9A* 42%

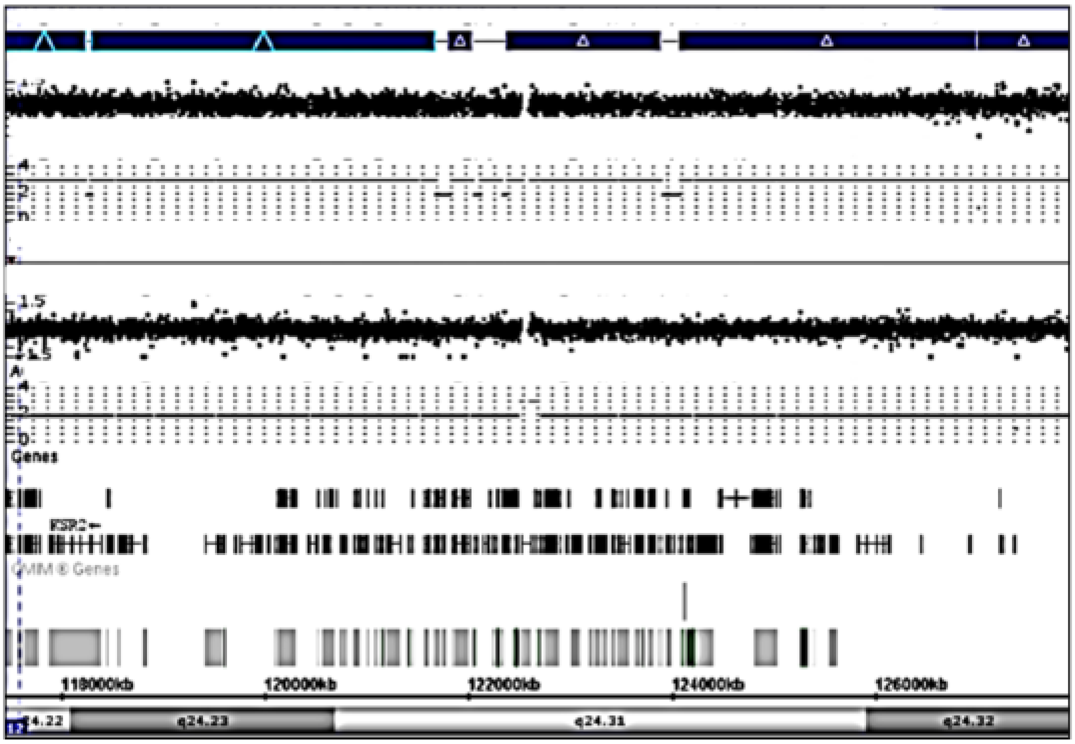

arr[hg19] 12q24.22q24.32(117,452,580-133,584,910)x2

Supplementary Figure 5E

EXCLI Journal 2022;21:117-143 – ISSN 1611-2156
Received: November 02, 2021, accepted: December 15, 2021, published: January 07, 2022

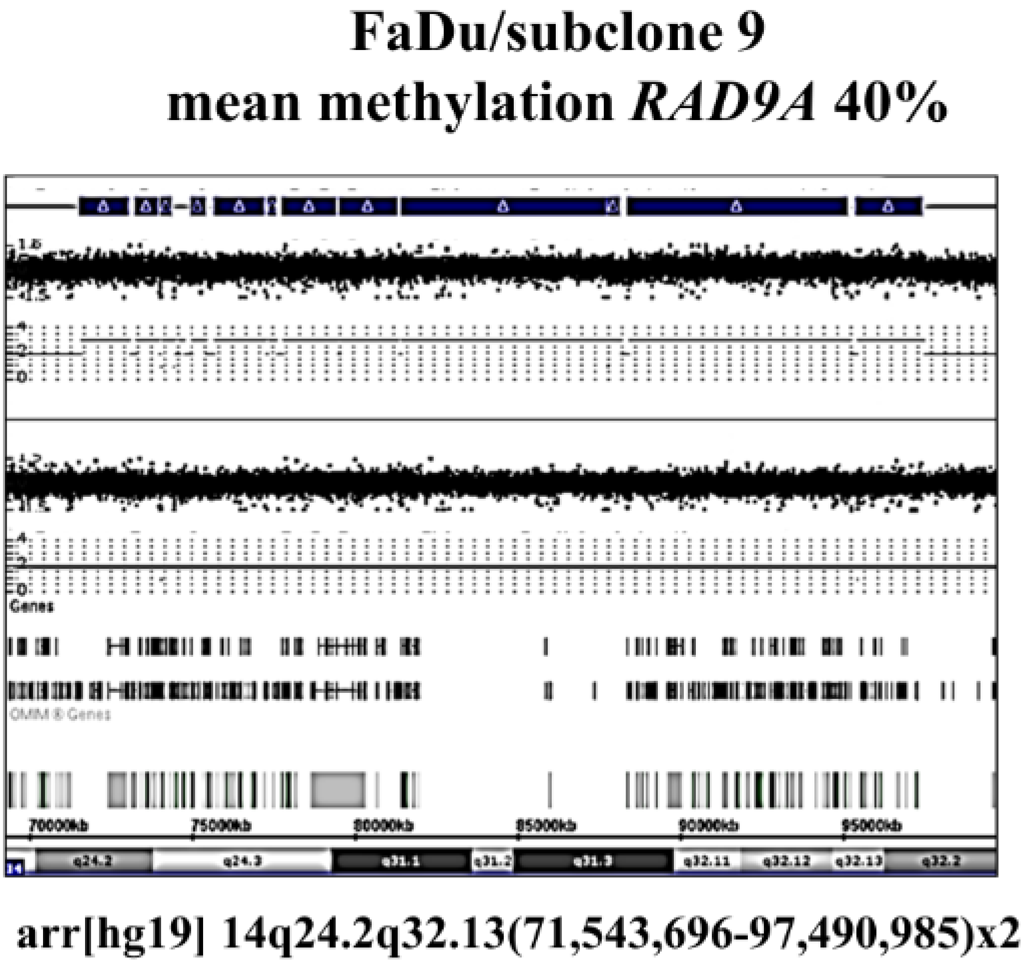

Supplementary Figure 5F

EXCLI Journal 2022;21:117-143 – ISSN 1611-2156
Received: November 02, 2021, accepted: December 15, 2021, published: January 07, 2022

Supplementary Table 1: List of PCR- and sequencing primer (5´-3´orientation) for bisulfite pyrosequencing

Gene	Chromosomal localization[a] (bp)	Amplicon size (bp)	Forward primer	Reverse primer	Sequencing primer	No. of CpG sites	Reference
BRCA1	Chr17: 43,125,274-43,125,506	232	ATTTAGAGTAG-AGGGTGAAGG	*TCTATCCCTCCCATCCTCTAATT	TGGGTGGTTAATTTAGAGT	5	Galetzka et al., 2012
CDKN2A	Chr9: 21,974,960-21,975,129	169	GGTTGTTTTYGGTT-GGTGTTTT	*ACCCTATCCCTCA-AATCCTCTAAAA	TTTTTGTTTG-GAAAGAT	2	Feng et al., 2007
TP53	Chr17: 7,674,136 - 7,674,298	162	*TTTTTTAGGTTGGTTT-TGATTGTA	AAAACACAACAAACCAA-TATACA	TAATAATAAAAATAAACCTC	2	Designed for this study (exon 6)
APC (part a) Promoter 1A	Chr5: 112,737,678-112,737,871	193	*GGTTAGGGTTAGG-TAGGTTGT	ACTACACCACTACAACCA-CATATC	CCACACCCAAC-CAA	7	Modified after Schatz et al., 2006
APC (part b*)* Promoter 1A	Chr5: 112,737,677-112,737,779	102	GGGTTAGGGTTAGGTAGGT	*TCCAAC-CAATTACACAAC-TACTTCTCTCT	AG-GGTTAGGTAGGTT	6	Modified after Schatz et al., 2006
RAD9A	Chr11: 67,392,508-67,392,610	102	GGTTTTTATGGG-GAAAGGAGG	*CCACAAACCCAAC-CCTCTAAC	TTTTATGGG-GAAAGGA	3	Modified after Cheng et al., 2005
EFNA5	Chr5: 107,670,853-107,670,957	104	GAGGGTTTAGGAG-GAAAAAGGAATTA	*CCCCCCAAACACAACTTAAC	AATTATAAGATG-GAGAGAAG	5	Kuang et al., 2008
FBN1	Chr15: 48,417,049-48,417,285	236	GTAGTAGGGTAG-AAATTTATAGT-TAGGTTT	*CCACTTTTATCCAC-CTATTTTCTAAT	ATTATAGTGTTT-TTTAAGAG	1	Flanagan et al., 2006

[a] according to Ensemble NCBI human assembly GRCh37 (Ensembl release 92).
* biotinylated

EXCLI Journal 2022;21:117-143 – ISSN 1611-2156
Received: November 02, 2021, accepted: December 15, 2021, published: January 07, 2022

References cited in Supplementary Table 1:

Cheng CK, Chow LWC, Loo WTY, Chan TK, Chan V. The cell cycle checkpoint gene Rad9 is a novel oncogene activated by 11q13 amplification and DNA methylation in breast cancer. Cancer Res. 2005;65: 8646–54. doi:10.1158/0008-5472.CAN-04-4243.

Feng W, Shen L, Wen S, Rosen DG, Jelinek J, Hu X, et al. Correlation between CpG methylation profiles and hormone receptor status in breast cancers. Breast Cancer Res. 2007;9:R57. doi:10.1186/bcr1762.

Flanagan JM, Popendikyte V, Pozdniakovaite N, Sobolev M, Assadzadeh A, Schumacher A, et al. Intra- and interindividual epigenetic variation in human germ cells. Am J Hum Genet. 2006;79:67–84.

Galetzka D, Hansmann T, El Hajj N, Weis E, Irmscher B, Ludwig M, et al. Monozygotic twins discordant for constitutive BRCA1 promoter methylation, childhood cancer and secondary cancer. Epigenetics. 2012;7:47–54. doi:10.4161/epi.7.1.18814.

Kuang S-Q, Tong W-G, Yang H, Lin W, Lee MK, Fang ZH, et al. Genome-wide identification of aberrantly methylated promoter associated CpG islands in acute lymphocytic leukemia. Leukemia. 2008;22: 1529–38. doi:10.1038/leu.2008.130.

Schatz P, Distler J, Berlin K, Schuster M. Novel method for high throughput DNA methylation marker evaluation using PNA-probe library hybridization and MALDI-TOF detection. Nucleic Acids Res. 2006;34: e59. doi:10.1093/nar/gkl218.

EXCLI Journal 2022;21:117-143 – ISSN 1611-2156
Received: November 02, 2021, accepted: December 15, 2021, published: January 07, 2022

Supplementary Table 2: Primers (5´-3´orientation) for deep bisulfite sequencing

Gene	Primer	Sequence (5′ to 3′)	CpG No.	Chromosomal localization (bp)	Amplicon length
APC	Forward	ACACTCTTTCCCTACACGACGCTCTT-CCGATCTGGTTAGGGTTAGGTAGGTTGT	16	Chr5:112,737,678-112,737,871	193bp
	Reverse	GTGACTGGAGTTCAGACGTGTGCTCTT-CCGATCTACTACACCACTACAACCACATATC			
CDKN2A	Forward	ACACTCTTTCCCTACACGACGCTCTT-CCGATCTGGTTGTTTT**Y**GGTTGGTGTTTT	10	Chr9:21,974,960-21,975,129	169bp
	Reverse	GTGACTGGAGTTCAGACGTGTGCTCTT-CCGATCTACCCTATCCCTCAAATCCTCTAAAA			
RAD9A	Forward	ACACTCTTTCCCTACACGACGCTCTT-CCGATCTGGTTTTTATGGGGAAAGGAGG	3	Chr11:67,392,508-67,392,610	102bp
	Reverse	GTGACTGGAGTTCAGACGTGTGCTCTT-CCGATCTCCACAAACCCAACCCTCTAAC			
TP53	Forward	ACACTCTTTCCCTACACGACGCTCTTCCGATCTTT-TTTTAGGTTGGTTTTGATTGTA	2	Chr17:7,674,136-7,674,298	162bp
	Reverse	GTGACTGGAGTTCAGACGTGTGCTCTT-CCGATCTAAAACACAACAAACCAATATACA			

[a] according to Ensemble NCBI human assembly GRCh38 (Ensembl release 104).
